# Selective deoxygenation of nitrate to nitrosyl using trivalent chromium and the Mashima reagent: reductive silylation†
†Electronic supplementary information (ESI) available: NMR, MS (ESI, APCI), FT-IR, EPR, XPS, CV, DFT, and crystal structure data for H_2_LCr(NO_3_)_2_(NO). CCDC 1848665. For ESI and crystallographic data in CIF or other electronic format see DOI: 10.1039/c8sc02979b


**DOI:** 10.1039/c8sc02979b

**Published:** 2018-10-16

**Authors:** Junghee Seo, Alyssa C. Cabelof, Chun-Hsing Chen, Kenneth G. Caulton

**Affiliations:** a Indiana University , Department of Chemistry , 800 E. Kirkwood Ave. , Bloomington , IN 47401 , USA . Email: caulton@indiana.edu

## Abstract

1,4-Bis(trimethylsilyl)-1,4-diaza-2,5-cyclohexadiene is an effective silyl transfer reagent towards the oxygen of nitrate coordinated to Cr(iii) in a pincer complex. Two nitrate oxygens are removed to give the 17 valence electron octahedral complex (H_2_L)Cr(NO_3_)_2_(NO). This is shown by a variety of spectroscopic methods, together with DFT, to be a Cr(i) complex with a linear CrNO unit. This work also identifies future applications of this reductive silylation process.

## Abstract 2

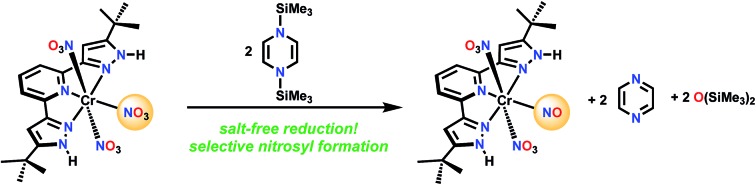
1,4-Bis(trimethylsilyl)-1,4-diaza-2,5-cyclohexadiene is an effective silyl transfer reagent towards the oxygen of nitrate coordinated to Cr(iii) in a pincer complex.

## Introduction

**Scheme 1:**
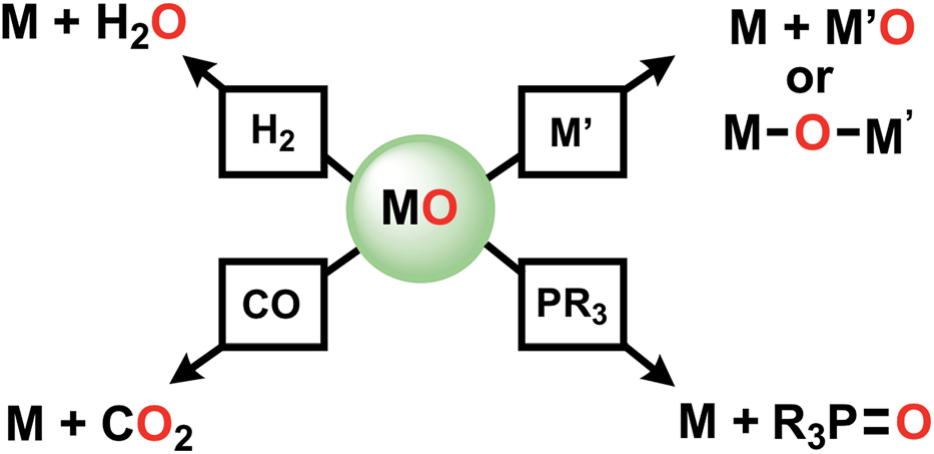
Precedent of oxygen atom transfer reactions.

This is the age of anthropogenic perturbation of the natural nitrogen cycle.^1^,^2^ Nitrogen is a limiting factor for agricultural production of food. Nitrogenous fertilizers, ammonia and ammonium nitrate, produced by the Haber–Bosch process, are crucial to meet that increasing food demand. An unintended consequence of meeting planetary food needs is increased runoff of NO_3_^–^, produced by soil bacteria, into ground water, which results in excessive growth of plants and algae (eutrophication) in poorly drained bodies of water. This problem demands new approaches to deoxygenation of nitrate. In contrast to deoxygenation of N^V^, abstraction of O atoms from a metal oxo complex can be accomplished by a low valent metal M′ (Scheme 1) *via* “oxygen atom transfer”, but this reaction can often merely form a bridging MOM′ complex.^3^–^6^ O atom abstraction can sometimes occur by two electron reduction by PR_3_, by CO, rarely and under forcing conditions with H_2_, or in more complicated transformations with boron hydrides.^7^

**Scheme 2:**
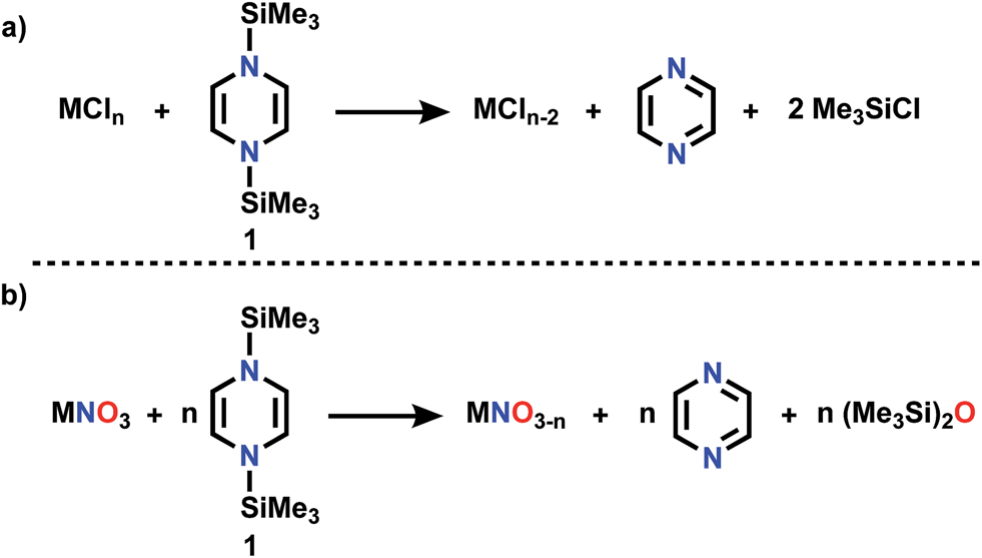
(a) Reductive silylation reaction using Mashima reagent (b) deoxygenation reaction using Mashima reagent.

On the other hand, our efforts to deoxygenate nitrate focus on the use of unconventional reagents. Kaim reported the electron rich character of silylated nitrogen heterocycles, generally 8 π electron molecules.^8^,^9^ These bis-silylated nonaromatic reagents were shown by Mashima and Tsurugi to reduce metal in MCl_*n*_ by abstracting chlorine, based on favorable thermodynamics of forming the Si–Cl bond and the energetic benefit of aromatization of the ring.^10^ We focus here on 1,4-bis(trimethylsilyl)-1,4-diaza-2,5-cyclohexadiene (Mashima reagent, **1**, Scheme 2). In reagent **1**, the Si–N bond dissociation energy should be weak, just as the doubly allylic C–H bonds in 1,4-cyclohexadiene are. Both nonmetal products, pyrazine and Me_3_SiCl, are volatile and thus readily removed and so this is termed by the discoverers as a “salt-free reduction route;” this is in contrast to elemental metal reductants, where the resulting oxidized metal halide can be challenging to remove. Subsequent reports have shown other applications including reduction of 4d and highly electropositive metals (niobium),^11^ dechlorination of geminal dihalides to olefins,^12^ and double silylation of metal oxides MO_*n*_ to yield reduced metal oxide.^13^,^14^ The method has also been applied in main group reductions^15^ and nanoparticle synthesis.^16^

Dechlorination (MCl_*n*_ → MCl_*n*–1_) (Scheme 2a) is a single electron reduction, but the Mashima reagent has special appeal for deoxygenation (Scheme 2b), since it appears to have the ability to accomplish a 2-electron reduction in a single molecular collision, a rare capacity among reductants. We report here new applications of reductive silylation: deoxygenation of coordinated NO_*x*_^–^ of relevance to remediation of nitrate-based eutrophication.^17^–^22^

## Results and discussion

**Scheme 3:**
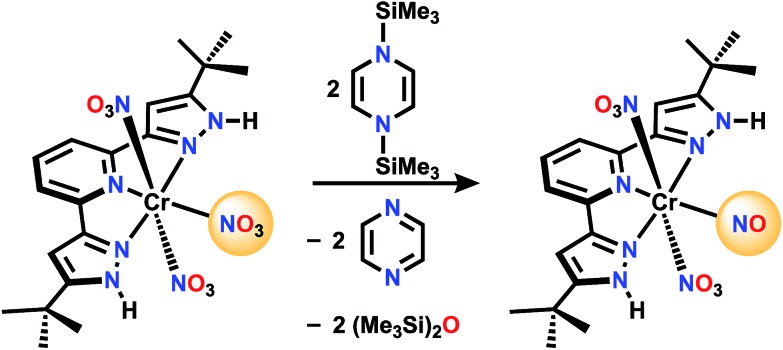
Reaction of (H_2_L)Cr(NO_3_)_3_ with the Mashima reagent.

For comparison, there is no reaction between [Ph_3_PNPPh_3_]NO_3_ and **1** in CD_2_Cl_2_ even after 72 h at 50 °C. A nitrate complex containing bis(pyrazol-3-yl)pyridine (H_2_L) ligand (Scheme 3), was chosen to be proton responsive, redox active, and carry useful ^1^H NMR spectroscopic reporter substituents for paramagnetic species. Reaction of (H_2_L)Cr(NO_3_)_3_ with **1** in CH_2_Cl_2_ at a 1 : 3 mole ratio is complete within 5 minutes, and the product shows a ^1^H NMR spectrum consistent with equivalent pyrazole arms, showing a paramagnetically shifted ^*t*^Bu resonance along with the stoichiometric amount of byproducts, hexamethyldisiloxane and pyrazine (see ESI†). The IR spectrum shows a strong absorption at 1720 cm^–1^, possibly indicative of a nitrosyl. Absorptions are also seen at 1484, 1415, 1313, 1285, and 990 cm^–1^ which are attributed to NO stretching and bending motions, but are not uniquely diagnostic of NO_*x*_ formula. The ESI(–) and APCI(–) mass spectra both show an ion of formula LCr(N_2_O_4_)^–^, while both positive ion methods show (H_2_L)Cr(N_2_O_4_)^+^. Single crystal X-ray diffraction of crystals grown by vapor diffusion of cyclohexane into a saturated THF solution were found to have formula (H_2_L)Cr(NO_3_)_2_(NO), with NO *trans* to the pyridine N. This is therefore designated a {CrNO}^5^ configuration,^23^,^24^ which are relatively common S = 1/2 species (*e.g.* Cr(NO)L_5_^2+^ where L = NH_3_, H_2_O or DMSO).^25^–^34^ Another analog is (picolinate) Cr(NO)(H_2_O)_2_, synthesized in a completely different way, from Cr^VI^ and reductant NH_2_OH.^35^,^36^ In those cases the Cr/L distance *trans* to the nitrosyl is always longer than those Cr/L *cis* to NO, consistent with a strong *trans* influence of the nitrosyl. In all cases, the CrNO^2+^ unit is linear, consistent with NO^+^ assignment, hence the product here is low spin Cr(i). This is consistent with 5 electrons in the t_2g_ derived orbitals, which maximizes back donation to NO^+^ in contrast to what would be true if any electrons populated the e_g_-derived orbital in a high spin alternative. The species LCr(N_2_O_4_)^–^ observed by mass spectrometry is therefore the result of loss of two protons and one weakly bound nitrate in the ionization process, to form species LCr(NO_3_)(NO)^–^.

**Fig. 1:**
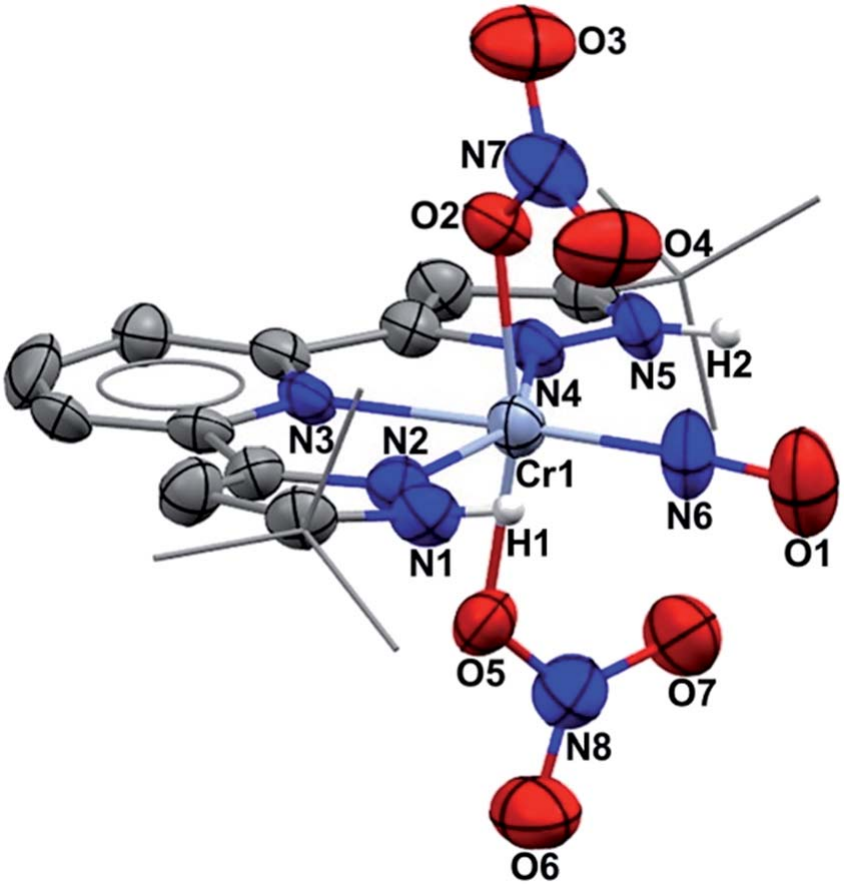
Mercury drawing (50% probabilities) of (H_2_L)Cr(NO_3_)_2_(NO), showing selected atom labelling. Unlabeled atoms are carbon and THF molecules hydrogen bonding to both NH protons are omitted, for clarity. Selected structural parameters (Å): Cr1–O2, 2.017(6); Cr1–O5, 2.003(6); Cr1–N2, 2.075(7); Cr1–N3, 2.078(5); Cr1–N4, 2.074(6); Cr1–N6, 1.724(6); O1–N6, 1.160(9).

Single crystal X-ray diffraction (Fig. 1) shows an octahedral structure with nitrosyl *trans* to pyridine nitrogen. NH protons do not hydrogen bond intramolecularly to nitrate oxygen, but instead to two THF molecules (O/N = 2.77 Å) which co-crystallize (see ESI†). The nitrate ligands rotate in opposite directions (yielding idealized molecular C_2_ symmetry), achieving O/N *β*(pz) distances of 3.06 Å. In fact those oxygens have approaches in the narrow range of 2.898–3.100 to both pyrazole nitrogens and the nitrosyl nitrogen. The Cr/NO distance is short (1.724(6) Å), as is the NO distance (1.160(9) Å). The nitrate ONO angles lie within the range 116.6–124.0° and the CrON angles are both 126.5°, consistent with sp^2^ hybridization. Binding nitrate to Cr here lengthens the N–OCr distance to 1.296 Å from the other four nitrate NO values of 1.219–1.251 Å; these are all longer than the triple bond in the nitrosyl. The exceptional result here is that four electron reduction of (H_2_L)Cr(NO_3_)_3_ occurs entirely on one nitrate ligand, not distributed over two. While this might first appear to mean production of NO^–^ (NO_3_^–^ → NO^–^ + 2 “O”), this undergoes intramolecular two electron reorganization with initially Cr(iii) to yield the observed [Cr^I^(NO^+^)]^2+^ product. Cr^I^ is an unusual oxidation state, and (H_2_L)Cr(NO_3_)_2_(NO) is a 17 valence electron species, but this deficit from 18 electron count follows due to the absence of any single electron reductant in the synthesis.

The XPS spectrum of (H_2_L)Cr(NO_3_)_2_(NO) was obtained to evaluate the resolution of this technique as well as its utility for obtaining atom ratios, including oxygen. Four samples were assayed to establish reproducibility. The measured Cr 2p_3/2_ binding energy was 577.0 eV, consistent with an oxidation state below 3^+^.^37^ The N 1s region resolved three types of nitrogen, with populations 2 : 1 : 5, consistent with nitrate (406.6 eV), nitrosyl (402.1 eV) and pincer ligand (400.2 eV). There was no resolution of the three types of oxygen, but the atom ratios of Cr : N : O were within 10% of the theoretical values 1 : 8 : 7.

**Fig. 2:**
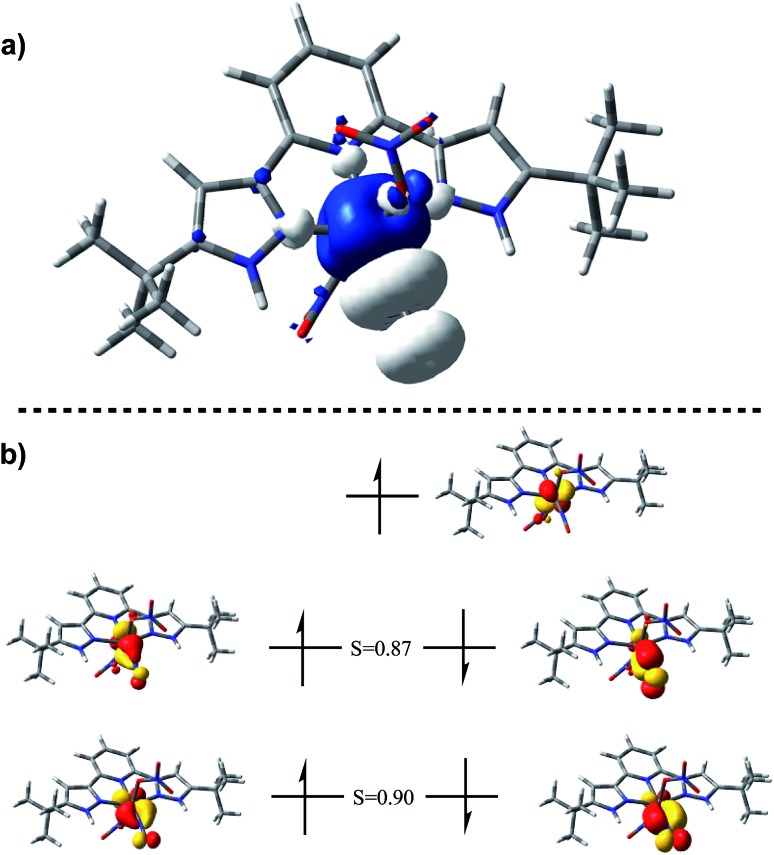
(a) Spin density plot for (H_2_L)Cr(NO_3_)_2_(NO) plotted on an isosurface value of 0.002 au. (b) Corresponding orbital analysis for (H_2_L)Cr(NO_3_)_2_(NO) plotted on an isosurface value of 0.05 au.

The EPR spectrum of (H_2_L)Cr(NO_3_)_2_(NO) shows a typical pattern^38^–^40^ for an S = 1/2 species, including satellites for A(^53^Cr) = 13.4 × 10^–4^ cm^–1^. In a THF solution at 25 °C, there is no resolved coupling to any ligand ^14^N, consistent with A(^14^N) < 7 × 10^–4^ cm^–1^, as well as consistent with the negligible nitrogen character in the calculated SOMO (Fig. 2b).

DFT calculations (BP86/6-311G(d)) on (H_2_L)Cr(NO_3_)_2_(NO) were used to establish the distribution of electrons within the {CrNO}^5^ unit. The calculated NO stretching frequency is 1746 cm^–1^, in satisfactory agreement with the experimental value of 1720 cm^–1^; both experimentally and computationally, this vibration is the most intense of all modes. The calculated nitrosyl complex has structural parameters in full agreement with the crystallographic ones. Mulliken spin densities are +2.2e on Cr, –0.69e on nitrosyl N and –0.50e on nitrosyl O, then negligible elsewhere. [see ESI†]. These are likewise consistent with the spin density plot in Fig. 2a.

Corresponding orbitals^41^ from an unrestricted calculation (Fig. 2b) show five electrons located in primarily t_2g_-derived Cr d orbitals, with high *α*/*β* overlap values. This is consistent with four paired electrons in orbitals of π symmetry with respect to the CrNO line, each of these have some 
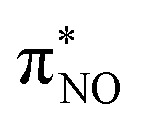
 character, indicative of back donation from metal to NO^+^. This also shows a SOMO which is on Cr, and of delta symmetry with respect to the NO, hence having no NO character. There is no spin density on H_2_L or on nitrate. Overall, this is consistent with the d^5^ configuration of Cr^I^ with significant back-donation into the nitrosyl π*. These results are consistent with EPR parameters and DFT results for analogous CrL_5_(NO) species.^25^–^39^

**Scheme 4:**
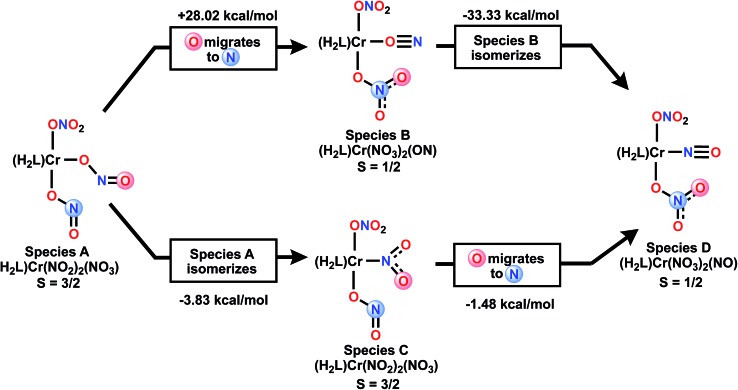
DFT free energies of two isomerization mechanisms.

Mechanistically, beyond the more obvious double deoxygenation^42^ of the same nitrate, other mechanisms can be considered. Scheme 4 envisions the alternative single deoxygenation of two nitrates, followed by two rearrangement steps of a bis-nitrito species which could then arrive at the observed product. We have used DFT to calculate the energies of various isomeric double deoxygenation products, to see which might be exergonic from a given isomer, *vs.* potentially highly endergonic. Species **A** and **C** are S = 3/2 and **B** and **D** are S = 1/2. Of special note in Scheme 4 is the CrON “isonitrosyl” species **B**^43^ which is found at extremely high energy, and thus not mechanistically relevant. For additional discussion of the electronic structure of this species, see ESI.† DFT calculations show that S = 3/2 states of the two species **A** and **C** are nearly isoenergetic (nitro isomer is 3.8 kcal mol^–1^ more stable), so interconversion of these is expected to be facile.

In order to quantitate the degree to which trading NO for SiO bonds yields favorable thermodynamics, we calculated the energy for the double deoxygenation reaction of H_2_LCr(NO_3_)_3_ to H_2_LCr(NO_2_)_2_(NO_3_) with two equivalents of the Mashima reagent [see ESI† for more details], with both reactant and product complexes in the S = 3/2 spin state. Calculations were done at the BP86/6-311G(d) level of theory, giving a reaction free energy of –158.1 kcal mol^–1^. This is the single deoxygenation of two nitrate groups, so the average of these, ∼–80 kcal mol^–1^, shows the impressive thermodynamic potential of the Mashima reagent for reductive silylation.

The 17 valence electron count of this species suggests it should be subject to reduction to yield an 18 electron {CrNO}^6^ complex. Indeed, a range of 18 valence electron [CrNO]^1+^ species have been isolated, and connected to the 17 electron class *via* cyclic voltammetry.^31^,^32^ CV of (H_2_L)Cr(NO_3_)_2_(NO) in THF with TBAPF_6_ as supporting electrolyte at 0.1 V s^–1^ in the range +0.7 to –3.0 V (*vs.* Fc/Fc+) shows a first reduction wave with *E*_pc_ = –2.18 V followed by two more current maxima at –2.55 and –2.90 V; there is no oxidation wave in that range. At sweep rates up to 1 V s^–1^, even the least negative of these reduction waves fails to show reversibility. This shows that one electron reduction occurs, but follow-up processes cause chemical change on the CV timescale, leaving no chance for reoxidation of transient (H_2_L)Cr(NO_3_)_2_(NO)^–^. Given the reducible protons, as well as the pentavalent nitrogen in this anion, redox chemistry is a likely cause of the CV irreversibility. In any event, the neutral molecule is clearly established to have an orbital available for accepting an electron.

## Conclusions

This work begins to show the potential of the Mashima reagent for deoxygenation of high valent nitrogen in coordinated nitrate under mild conditions. The activating effect of trivalent chromium is clearly important, as shown here by the lack of deoxygenation of free nitrate. Deoxygenation occurs twice to finally take nitrate to the coordinated nitrosyl oxidation level. The selective conversion of (H_2_L)Cr(NO_3_)_3_ to (H_2_L)Cr(NO_3_)_2_(NO) shows that the weakly acidic NH protons are not reactive towards the silyl reagent; the reagent has oxophilic selectivity. The results reported here show that deoxygenation of nitrate is favored compared to conversion of (H_2_L)Cr^III^(NO_3_)_3_ with **1** to (H_2_L)Cr^II^(NO_3_)_2_, pyrazine and (Me_3_Si)NO_3_. Further development of this reagent will benefit from better understanding of the mechanism of the reaction: electron transfer, silyl radical transfer, or concerted transfer of two silyl groups in a single molecular collision.^44^–^46^ Further topics with great potential include the generality of the Mashima reagent to deoxygenate nitrite, one example of which is demonstrated here. Another step worth exploration is its potential for deoxygenation of nitrosyl itself, as well as a determination of whether the resulting metal nitrides could be converted to silyl imines and even the bis-silyl amide ligand, N(SiMe_3_)_2_, these being one- and two-electron reductions of the metal, respectively. More broadly still, can the Mashima reagent deoxygenate other oxyanions: perchlorate, sulfite, phosphite, and even carbonate?

## Conflicts of interest

There are no conflicts to declare.

## Supplementary Material

Supplementary information

Crystal structure data
