# Supplementary material for: Selective deoxygenation of nitrate to nitrosyl using trivalent chromium and the Mashima reagent: reductive silylation
Source: Chem Sci. 2018 Oct 16;10(2):475–9. doi: 10.1039/c8sc02979b (PMC6335631; doi:10.1039/c8sc02979b)
Supplement: Supplementary file 1 [file SC-010-C8SC02979B-s001.pdf]

Electronic Supplementary Material (ESI) for Chemical Science.
This journal is © The Royal Society of Chemistry 2018

Supporting information for:

**Selective Deoxygenation of Nitrate to Nitrosyl using Trivalent Chromium and the Mashima Reagent: Reductive Silylation**

Junghee Seo, Alyssa C. Cabelof, Chun–Hsing Chen and Kenneth G. Caulton*

**Table of Contents**

**Experimental** **S2-S3**
- General — S2
- Synthesis of $(H_2L)Cr(NO_3)_3$ — S2
- Synthesis of $(H_2L)Cr(NO_3)_2(NO)$ — S2
- Titration of $(H_2L)Cr(NO_3)_2(NO)$ with **1** — S2
- Reaction of $[Ph_3PNPPh_3]^+NO_3^-$ with **1** — S3

**Spectral Data** **S3-S6**
- Figure S1: $^1H$ NMR of $H_2LCr(NO)(NO_3)_2$ — S3
- Figure S2: Progressive $^1H$ NMR spectra of titration of $H_2LCr(NO_3)_3$ with **1** — S4
- Figure S3: FT-IR spectrum of $H_2LCr(NO_3)_2(NO)$ — S4
- Figure S4: Mass spectra of $H_2LCr(NO_3)_2(NO)$ — S5
- Figure S5: EPR spectrum of $(H_2L)Cr(NO_3)_2(NO)$ — S6
- Figure S6: CV of $H_2LCr(NO_3)_2(NO)$ — S6

**XPS Data** **S7-S9**
- General details — S7
- Figure S7: XPS survey scan of $H_2LCr(NO_3)_2(NO)$ — S7
- Figure S8: XPS $Cr2p_{3/2 \text{ and } ½}$ spectrum — S8
- Figure S9: XPS N1s spectrum — S8
- Figure S10: XPS O1s spectrum — S9

**Crystallographic data** **S9-S11**
- General details — S9
- Data collection — S9
- Structure solution and refinement — S9
- References — S10
- Figure S11: Mercury drawing of $(H_2L)Cr(NO_3)_2(NO)$ — S10
- Table S1: Bond length comparison of $(H_2L)Cr(NO_3)_2(NO)$ and $(H_2L)Cr(NO_3)_3$ — S11

**Computational details** **S11-S24**
- General — S11
- References — S11
- Figure S12: Labelling scheme for all calculated species — S12
- Isonitrosyl analysis — S12
- Figure S13: Spin density plot for species B — S12
- Figure S14: Corresponding orbital analysis of species B — S12
- Table S2: Mulliken spin densities for species B — S13
- Table S3: Mulliken spin densities for species D — S13
- Table S4: Computed IR data — S14
- Figure S15: Overall thermodynamics for double deoxygantation of $(H_2L)Cr(NO_3)_3$ — S14
- Figure S16: Bond length comparisons for double and triple zeta calcs — S14
- Table S6: Cartesian coordinates for all optimized species — S15
- Table S5: thermodynamic data for all optimized species — S24

## Experimental

**General.** NMR spectra were obtained at 25 °C on a Varian Inova 400 MHz. $^1$H NMR of paramagnetic compounds were obtained with relaxation delay at 0.1 sec and at least 1024 scans per spectrum. IR spectra were obtained on a Nicolet 6700. Mass spectra were obtained using an Agilent 1200 HPLC-6130 MSD with ESI and APCI sources. All experiments were carried out in the glovebox (MBRAUN Labmaster 130) under ultra-high nitrogen or by applying the Schlenk techniques under an argon atmosphere. Solvents were purchased from Sigma Aldrich, Alfa Aesar, and other suppliers. All solvents and deuterated solvents were distilled by drying over drying agents such as $CaH_2$ or $P_2O_5$, or purified using Innovative Technology Inc's SPS-400 PureSolv solvent system prior to use. Glassware was oven-dried overnight or flame dried prior to use. Other reagents were commercial and used as received without further purification. 1,4-bis(trimethylsilyl)-1,4-diaza-2,5-cyclohexadiene was synthesized according to literature[1].

(1) R. A. Sulzbach and A. F. M. Iqbal, *Angew. Chem. Int. Ed.*, 1971, **10**, 127-127.

**Synthesis of $(H_2L)Cr(NO_3)_3$.** In a 20 mL blackened vial, $(H_2L)CrCl_3$ (75 mg, 0.156 mmol) was suspended in 5 mL of MeCN and allowed to stir for 5 minutes. To this blue-green slurry, $AgNO_3$ (85 mg, 0.499 mmol) was added as a solid. The mixture was allowed to stir for 1 hour. The resulting pink-purple suspension (a mixture of purple solution with white AgCl solid) was filtered through Celite. The purple filtrate was reduced in volume under vacuum to give a purple solid. This reaction was also carried out in $CH_2Cl_2$, which requires 5 hours of reaction time. Yield: 84 mg (97 %). **FT-IR (cm$^{-1}$):** 1507s (asym $\nu_{NO}$), 1302s (sym $\nu_{NO}$), 1276s (sym $\nu_{NO}$), 990m (bend, $_{NO2}$). **$^1$H NMR ($CD_2Cl_2$, 400 MHz, 298 K):** $\delta$ (ppm), 2.8 (tBu), −5.8 (ring H). **ESI-MS (−)** in THF: $[LCr(NO_3)_2]^-$ (calc. for $C_{19}H_{23}CrN_7O_6$ = 497.1, found, 497.1).

**Synthesis of $(H_2L)Cr(NO_3)_2(NO)$.** $(H_2L)Cr(NO_3)_3$ (25mg, 0.045 mmol) was suspended in 3 mL of $CH_2Cl_2$ and allowed to stir at room temperature for 10 minutes. To this pink suspension, a small excess of **1** (30 mg, 0.133 mmol) dissolved in 2 mL of $CH_2Cl_2$ was added slowly over the course of 2 minutes with an immediate but progressive color change from pink to dark green upon complete addition. This mixture was allowed to stir for 10 minutes then filtered over Celite to filter off a trace amount of black precipitate; this was found by XPS to contain negligible amount of Cr. The dark green solution was pumped to dryness to give a dark green solid. The dark green solid was washed with toluene to remove co-product, pyrazine, and dried under vacuum. Single crystals for X-ray diffraction were grown by vapor diffusion of cyclohexane into a saturated THF solution at 25 °C. Yield: 20 mg (0.038 mmol, 84%). **$^1$H NMR (400 MHz, $CD_2Cl_2$, 298K):** $\delta$ (ppm), 9.59 (1H), 2.37 (18H). **FT-IR (cm$^{-1}$):** 1720 ($\nu_{NO}$, nitrosyl), and the following, attributed to nitrate, which are absent for coordinated $H_2L$: 1484, 1415, 1313, 1285, 990. **ESI-MS (-) & APCI-MS (-)** in THF: $[LCrN_2O_4]^-$ (calc. for $C_{19}H_{23}CrN_7O_4$ = 465.1, found: 465.1). **ESI-MS (+) & APCI-MS (+)** in THF: $[H_2LCrN_2O_4]^+$ (calc. for $C_{19}H_{25}CrN_7O_4$ = 467.1, found: 467.1).

**$^1$H Monitoring a titration of 1 into $(H_2L)Cr(NO_3)_3$**

We explored a *stepwise* execution of the double deoxygenation. $^1$H NMR assay of addition of increasing equivalents (from 1:1 to 3:1) of a stock solution of **1** to $(H_2L)Cr(NO_3)_3$ in $CD_2Cl_2$ showed progressive disappearance of $(H_2L)Cr(NO_3)_3$ and growth of at least one intermediate, then finally, at 3:1 mole ratio, the first traces of unconsumed reagent **1**. At every step, the $(Me_3Si)_2O$ and pyrazine signal intensities grow in the expected stoichiometric ratio. The reaction was complete in less than ten minutes at each step, and a precipitate formed at the 2:1 stage dissolved, with additional color change, at the third addition; we did not further study the precipitate since our focus was on the nitrosyl product. The product at 3:1 mole ratio was confirmed to be $(H_2L)Cr(NO_3)_2(NO)$ by $^1$H NMR and IR spectroscopies and mass spectrometry. The need for modest excess of **1** (*vs.* eq. 1) is apparently related only to achieving a convenient rate, since 2:1 should be enough to reach the crystallographic product.

$(H_2L)Cr(NO_3)_3$ + 2 **1** $\rightarrow$ $(H_2L)Cr(NO_3)_2(NO)$ + 2 $(Me_3Si)_2O$ + 2 pyrazine (1)

$(H_2L)Cr(NO_3)_3$ (6mg, 0.0107 mmol) was suspended in 1mL of $CD_2Cl_2$ in a J Young tube, and was characterized with $^1H$ NMR. A stock solution of **1** (7.26 mg, 0.0321 mmol) in 1 mL $CD_2Cl_2$ was prepared and was added by syringe to the J Young tube stepwise in three portions. At the first addition, the mixture immediately turned to a green solution from purple-pink suspension. **$^1H$ NMR (400 MHz, $CD_2Cl_2$ 298K):** $\delta$ (ppm), 9.65 (1H, pyridine-para, product) 8.5 (pyrazine, co-product), 2.78 (<18H, $H_2L$-tBu, $(H_2L)Cr(NO_3)_3$), 2.34 ($H_2L$-tBu, intermediate), 0.06 ($(TMS)_2O$, co-product), -5.9 (<1H, pyridine-para, $H_2LCr(NO_3)_3$). At the second addition, an immediate color change to darker green, then an additional color change to light brown and finally precipitation after 10 minutes of reaction were observed. **$^1H$ NMR (400 MHz, $CD_2Cl_2$ 298K):** $\delta$ (ppm), 9.65 (1H, pyridine-para) 8.5 (pyrazine), 2.34 ($H_2L$-tBu, intermediate), 1.73 ($H_2L$-tBu, intermediate), 0.06 ($(TMS)_2O$, co-product). At the third addition, the precipitate re-dissolved and the solution turned green. **$^1H$ NMR (400 MHz, $CD_2Cl_2$ 298K):** $\delta$ (ppm), 9.65 (1H, pyridine-para, product) 8.5 (pyrazine, co-product), 4.5 (ring H-**1**) 2.42 (18H, $H_2L$-tBu, product), 0.41 (Methyl H-**1**), 0.06 ($(TMS)_2O$, co-product).

**Attempted reaction of $[Ph_3PNPPh_3]NO_3$ and 1.** 25 mg of $[Ph_3PNPPh_3]NO_3$ was added to a Teflon-closure NMR tube in $CD_2Cl_2$ giving a colorless solution. 1 equivalent of **1** was added, causing no color change. The reaction mixture was agitated for 72 hours, upon which no spectral changes were observed. The solution was heated to 50°C for a period of one week. No reaction occurred, as judged by the $^1H$ NMR resonances of both $[Ph_3PNPPh_3]NO_3$ and **1**.

<u>**Spectral Data**</u>

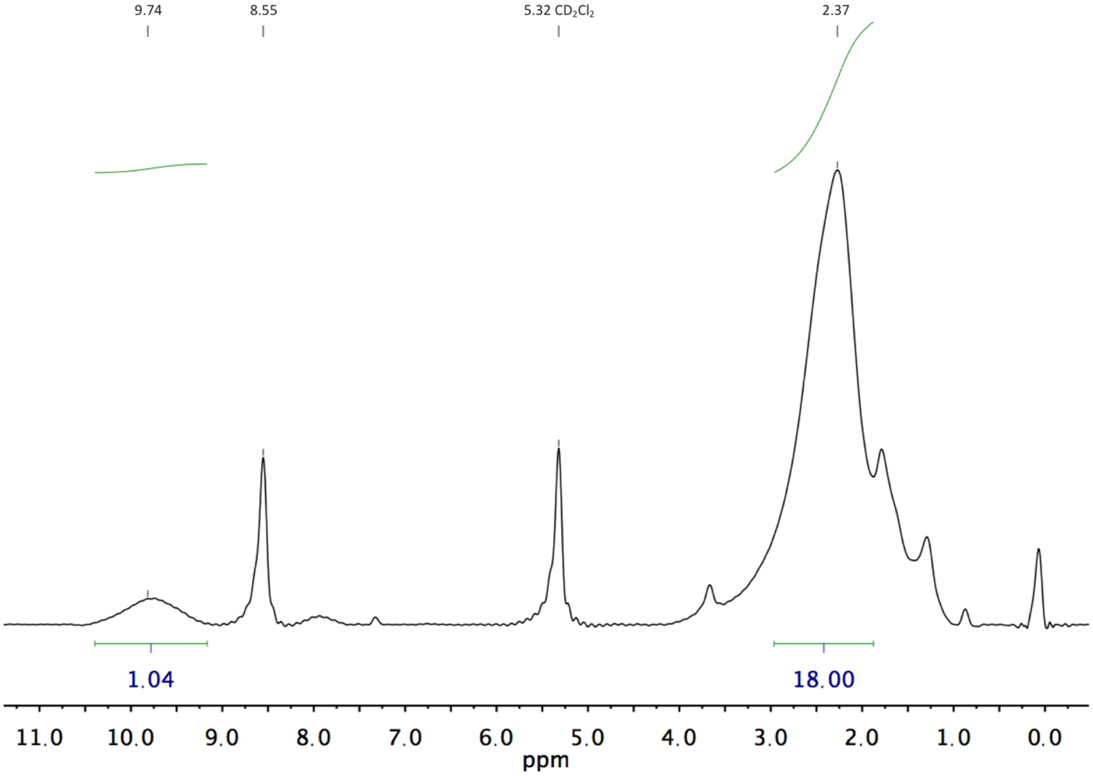


**Figure S1 .** $^1H$ NMR of $H_2LCr(NO_3)_2(NO)$ in $CD_2Cl_2$. Residual pyrazine gives signal at 8.55 ppm.

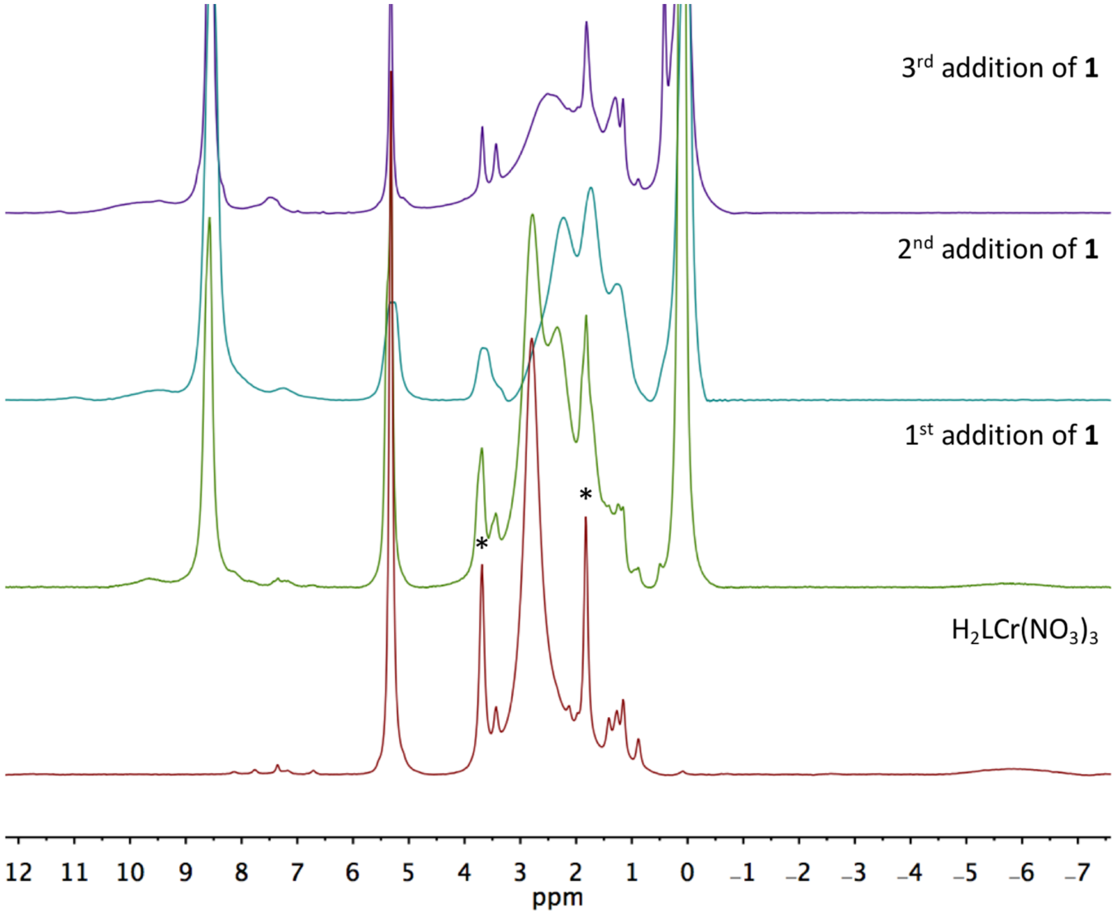


**Figure S2.** Progressive $^{1}H$ NMR spectra of titration of $H_2LCr(NO_3)_3$ with **1** in $CD_2Cl_2$. * = THF

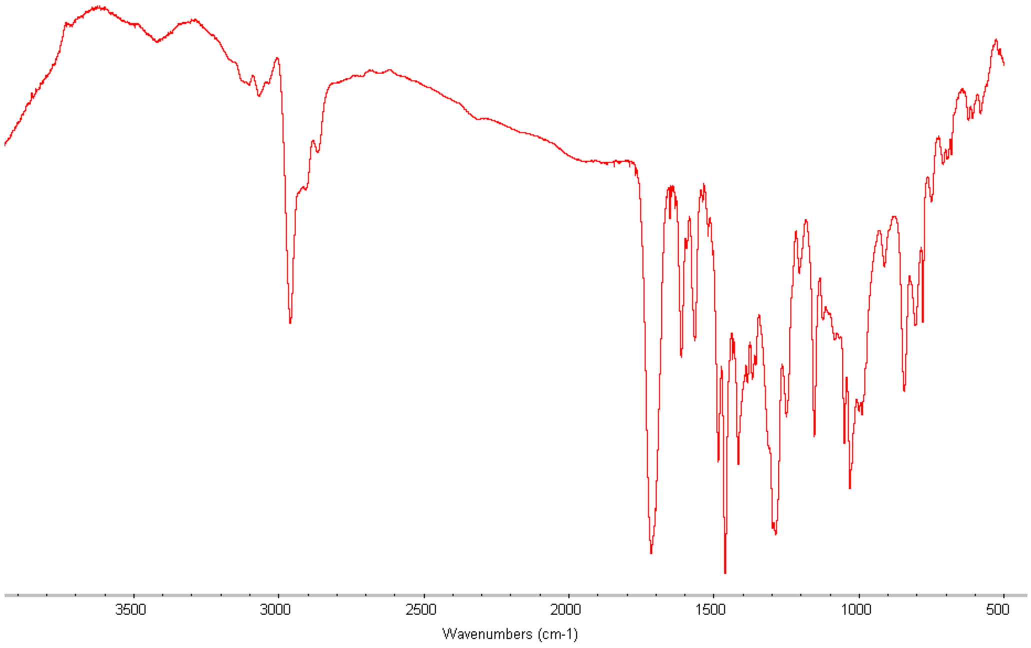


**Figure S3.** FT-IR of $H_2LCr(NO_3)_2(NO)$ on KBr disc.

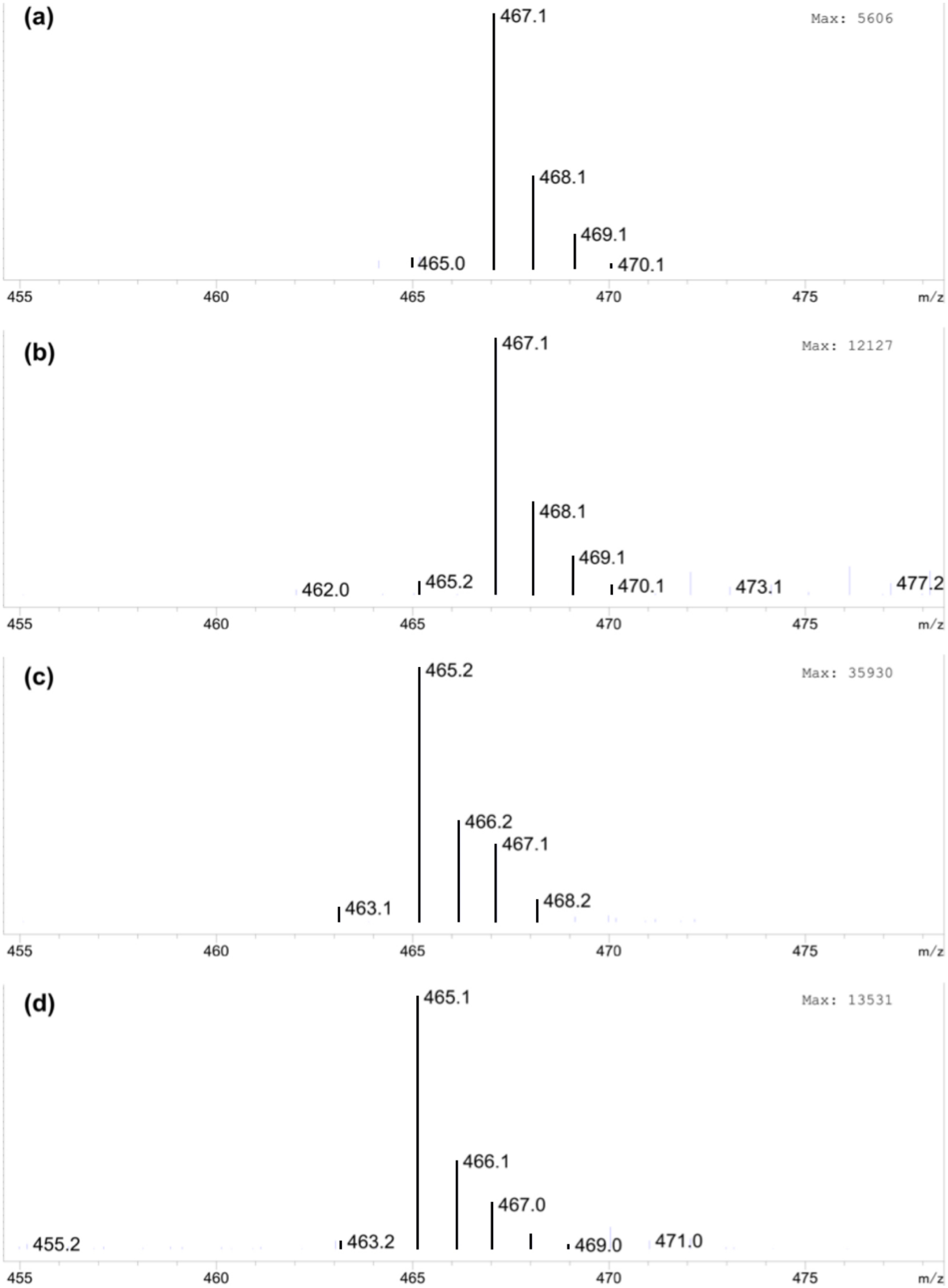


**Figure S4.** MS of $H_2LCr(NO_3)_2(NO)$ in THF: (a) ESI pos, (b) APCI pos, (c) ESI neg, (d) APCI neg.

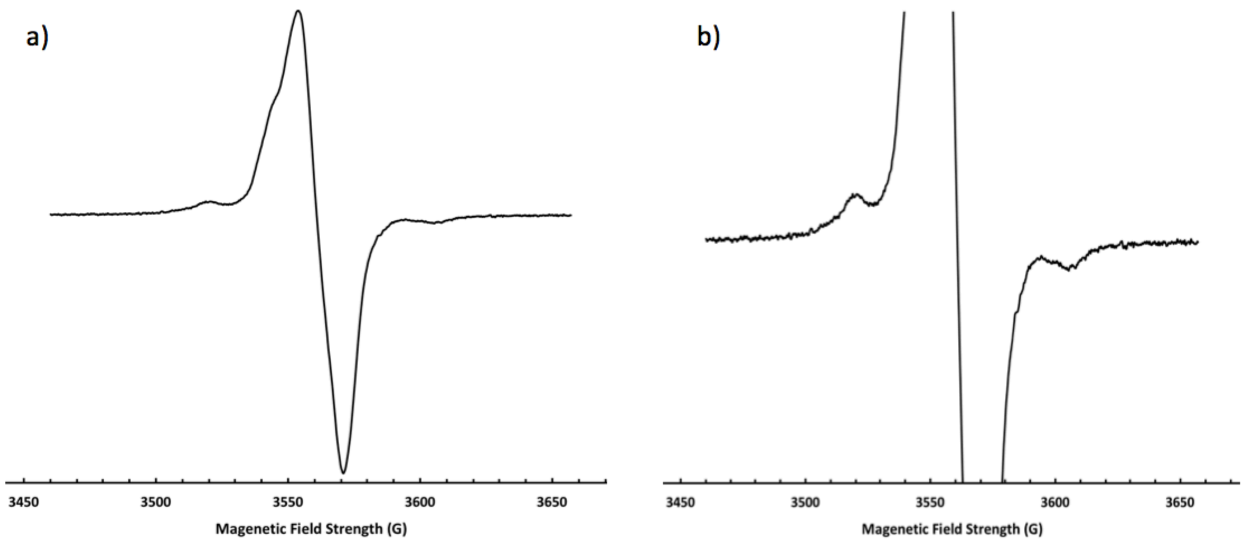


**Figure S5.** a) EPR spectrum of a 3.78 mM $(H_2L)Cr(NO_3)_2(NO)$ in THF at 298K. b) the blow-up about the center of the full spectrum. $g_{iso}$ = 1.970, $A_{iso}(^{53}Cr)$ = 0.00264 $cm^{-1}$, $A_{iso}(^{14}N)$ < 0.0007 $cm^{-1}$. Hyperfine constants are calculated using the relation A(in $cm^{-1}$) = A(in Gauss) x *g* x 4.67 x $10^{-5}$ $cm^{-1}$.

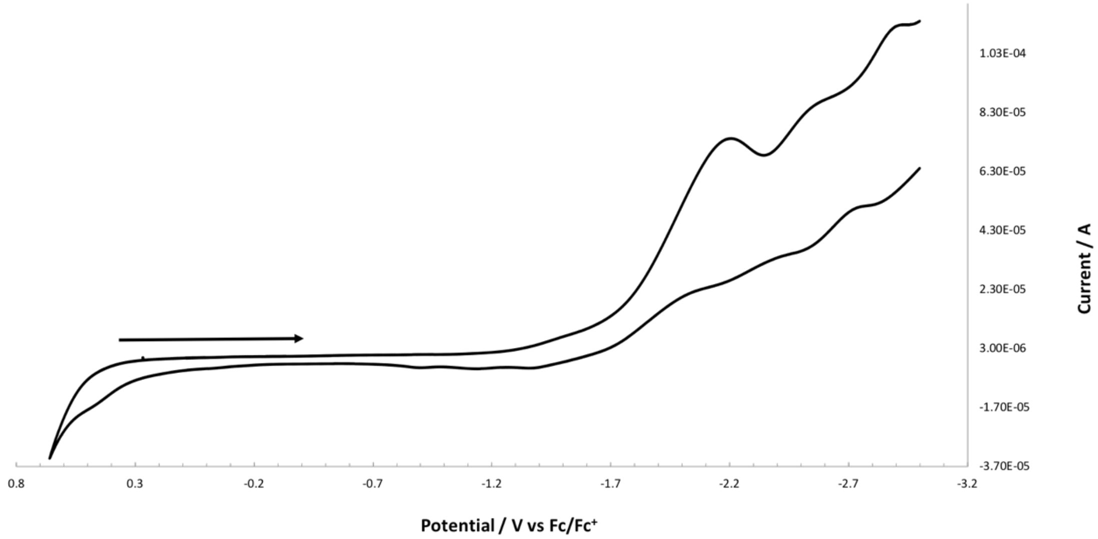


**Figure S6.** CV of 7.5 mM $H_2LCr(NO_3)_2(NO)$ in THF with 0.1 M $TBAPF_6$ as supporting electrolyte.

## XPS Data

**General.** Spectra were recorded on a PHI *Versa Probe* II instrument equipped with monochromatic Al K(alpha) source. Instrument base pressure was ca. 1.8 × $10^{-10}$ Torr. The X-ray power of 65 W at 15 kV was used for all experiments with 260 micron beam size at X-ray incidence and take off angle of 45°. The spectrometer dispersion was adjusted to give a BE of 284.8 eV for the C 1s line of adventitious (aliphatic) carbon present. The PHI dual beam charge neutralization system was used during all measurements. The high resolution Cr 2p, O 1s and C 1s spectra were taken with a minimum of 10 - 60 scans using a 0.1 eV step and 23.5 eV pass energy; the HR N 1s using 46.95 eV pass energy. Chloride and silver were absent from the samples studied. The ultimate *Versa Probe* II instrumental resolution was determined to be better than 0.125 eV using the Fermi edge of the valence band for metallic gold. The resolution with charge neutralization system was <0.68 eV FWHM on PET. All XPS spectra were recorded using PHI software *SmartSoft –XPS* v2.6.3.4 and processed using PHI *MultiPack* v9.3.0.3 and/or CasaXPS v.2.3.14. The XPS spectra were fitted using a combination of Gaussian and Lorentzian line shapes with 30-50% of Lorentzian contents. Shirley and/or iterated Shirley background was used to fit experimental data.

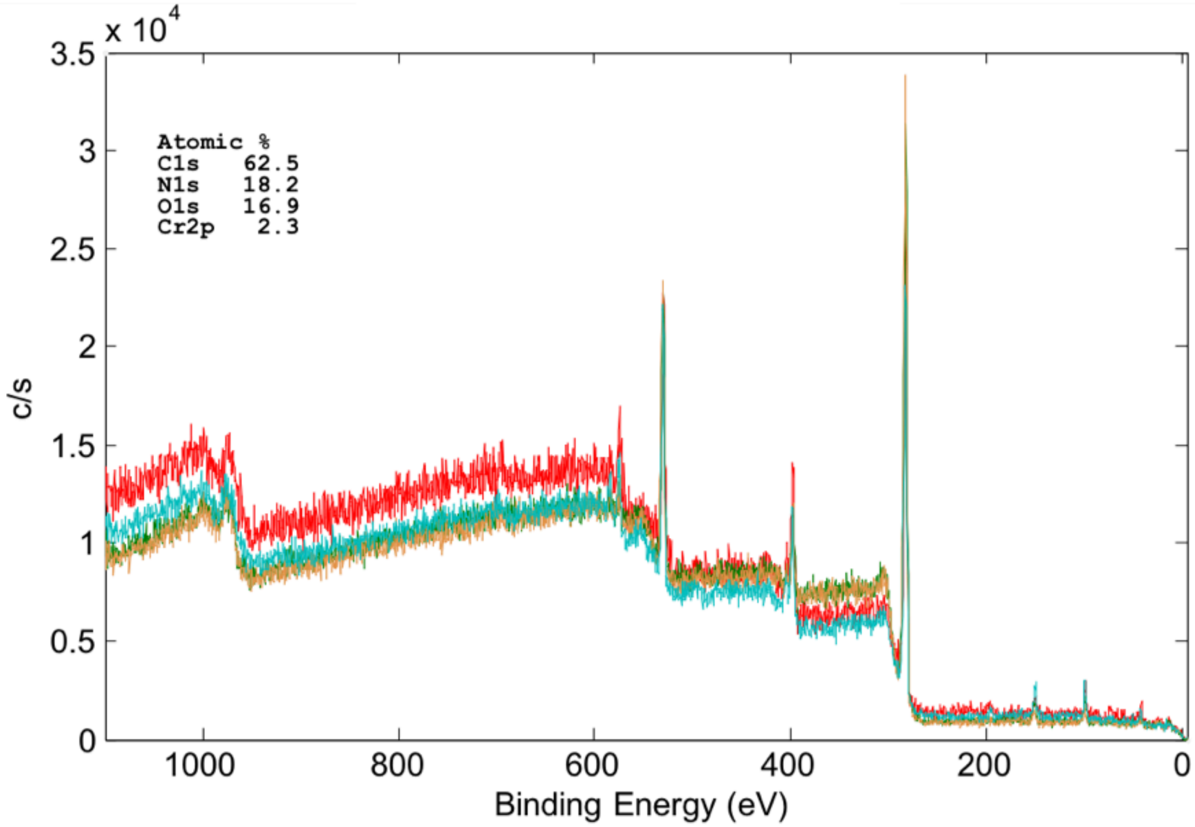


**Figure S7.** XPS survey scan of three different sample locations of $H_2LCr(NO_3)_2(NO)$, to assess spectral reproducibility.

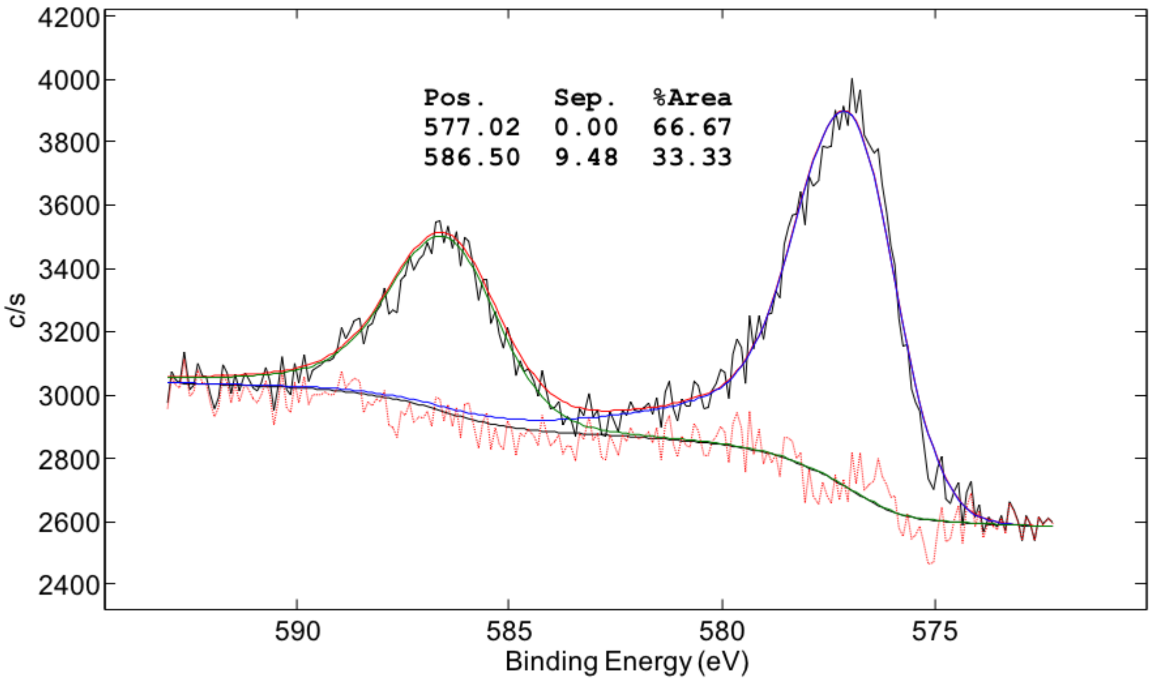


**Figure S8.** XPS Cr2p3/2 and ½ spectrum, showing binding energy indicative of Cr oxidation state below 3+.

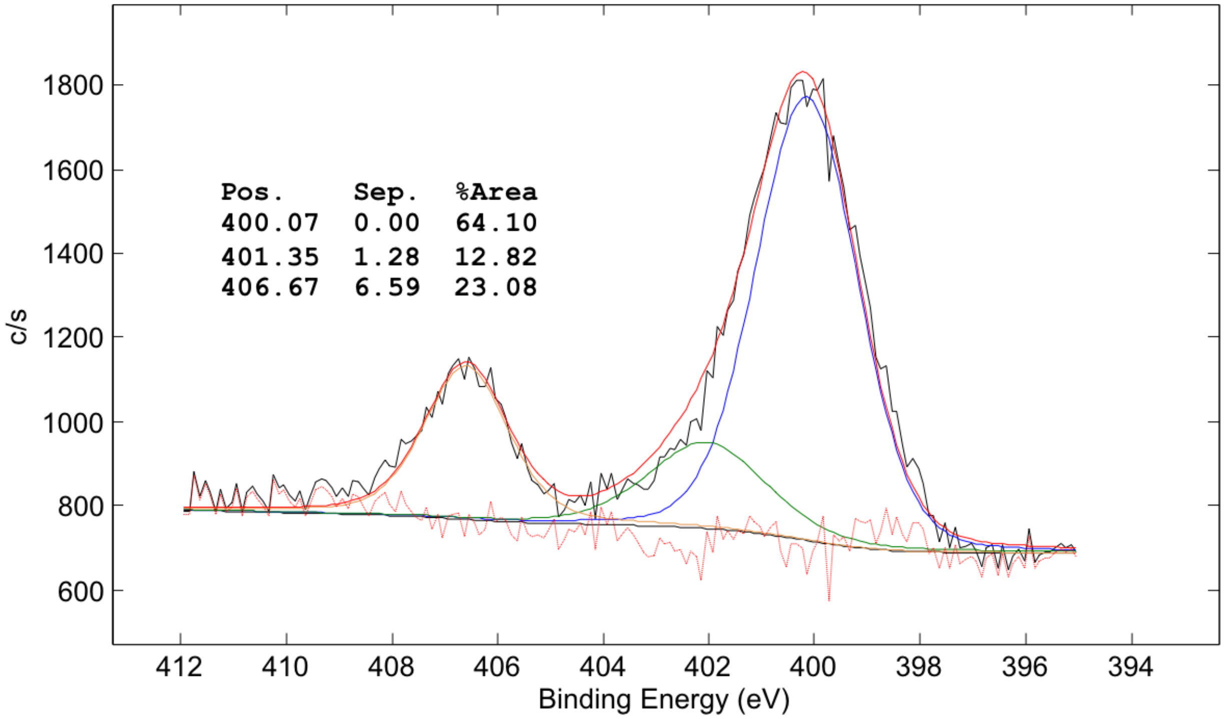


**Figure S9.** XPS N1s spectrum

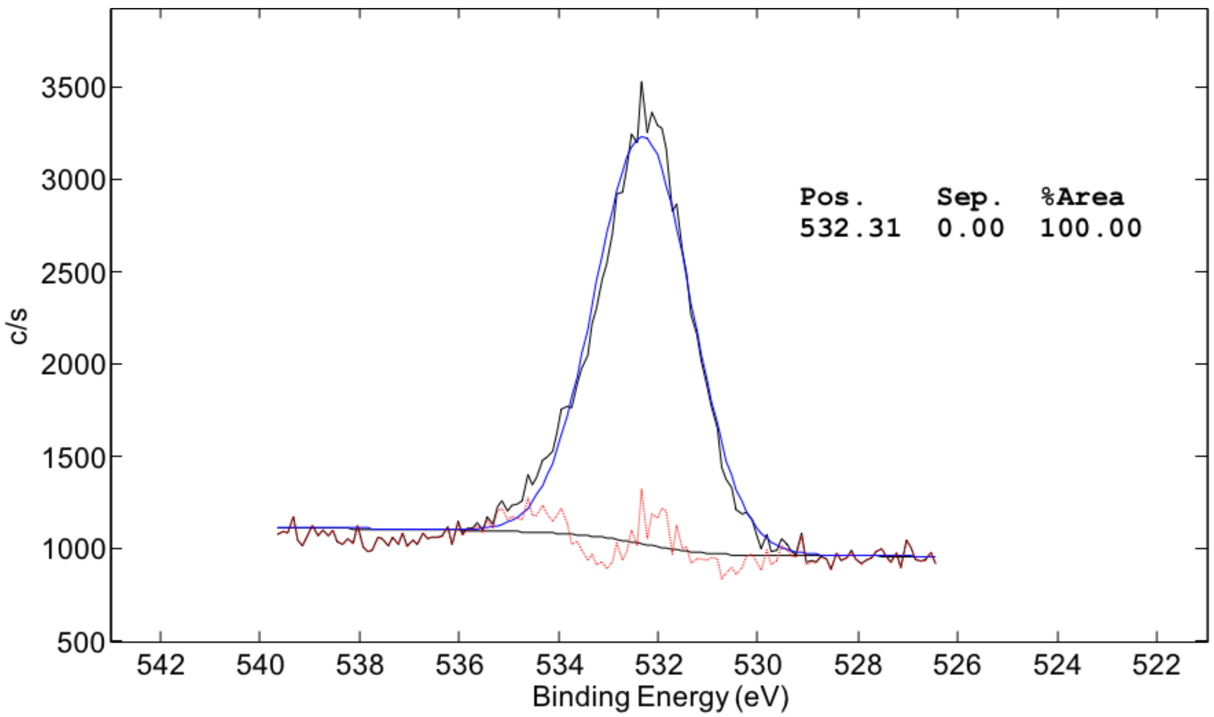


**Figure S10.** XPS O1s spectrum.

**Crystallographic Data.**

**General Details (S18001 Chen $H_2LCr(NO_3)_2(NO)$).** The sample was investigated with synchrotron radiation at the ChemMatCARS beamline, Advanced Photon Source, Argonne National Laboratory, Chicago, utilizing the SCrAPS program A yellow crystal (approximate dimensions 0.060 x 0.030 x 0.020 $mm^3$) was placed onto the tip of a glass capillary and mounted on a Huber three-circle diffractometer and measured at 100 K.
(http://www.iumsc.indiana.edu/projects/SCrAPS/index.html).

**Data collection.** The data collection was carried out using synchrotron radiation (λ = 0.41328, silicon 111 and 311 monochromators, two mirrors to exclude higher harmonics) with a frame time of 0.7 seconds and a detector distance of 5.0 cm. A randomly oriented region of reciprocal space was surveyed to the extent of hemispheres. Two major sections of frames were collected with 0.50º steps in φ and a detector position of 0° in 2θ. Data to a resolution of 0.84 Å were considered in the reduction. Final cell constants were calculated from the xyz centroids of 9300 strong reflections from the actual data collection after integration (SAINT).[1] The intensity data were corrected for absorption (SADABS).[2]

**Structure solution and refinement.** The space group *C*2/*c* was determined based on intensity statistics and systematic absences. The structure was solved using SHELX[3] and refined (full-matrix-least squares) using the Oxford University Crystals for Windows system.[4] The charge-flipping solution provided most non-hydrogen atoms from the E-map. Full-matrix least squares / difference Fourier cycles were performed, which located the remaining non-hydrogen atoms.
Structure S18001 diffracts weakly and contains pseudo-merohedral twin with twin law of the second

orientation [-1 0 -1  0 -1 0  0 0 1] and ROTAX[5] was used, resulting a drop in the R factor by 12%. Additional two-part disorder on one solvent THF was resolved successfully. The two-part disorder was modeled so the occupancies of the major and minor components summed to 1, with major component of the disorder refined with anisotropic displacement parameters, and minor component isotropically. All ordered non-hydrogen atoms were refined with anisotropic displacement parameters. The hydrogen atoms were placed in ideal positions and refined as riding atoms with. The final full matrix least squares refinement converged to R1 = 0.1283 and wR2 = 0.3578 ($F^2$, all data).

**References.**


1 SAINT, Bruker Analytical X-Ray Systems, Madison, WI, current version.

2 An empirical correction for absorption anisotropy, R. Blessing, Acta Cryst. A51, 33 - 38 (1995).

3 A short history of SHELX. G. M. Sheldrick, Acta Cryst. A64, 112 - 122 (2008)..

4 Betteridge, P. W.; Carruthers, J. R.; Cooper, R. I.; Prout, K.;  Watkin, D. J. J. Appl. Cryst. 2003, 36, 1487.

5 Cooper, R. I., Gould, R. O., Parsons, S. & Watkin, D. J. J. Appl. Cryst. 35, 168–174. 2002.


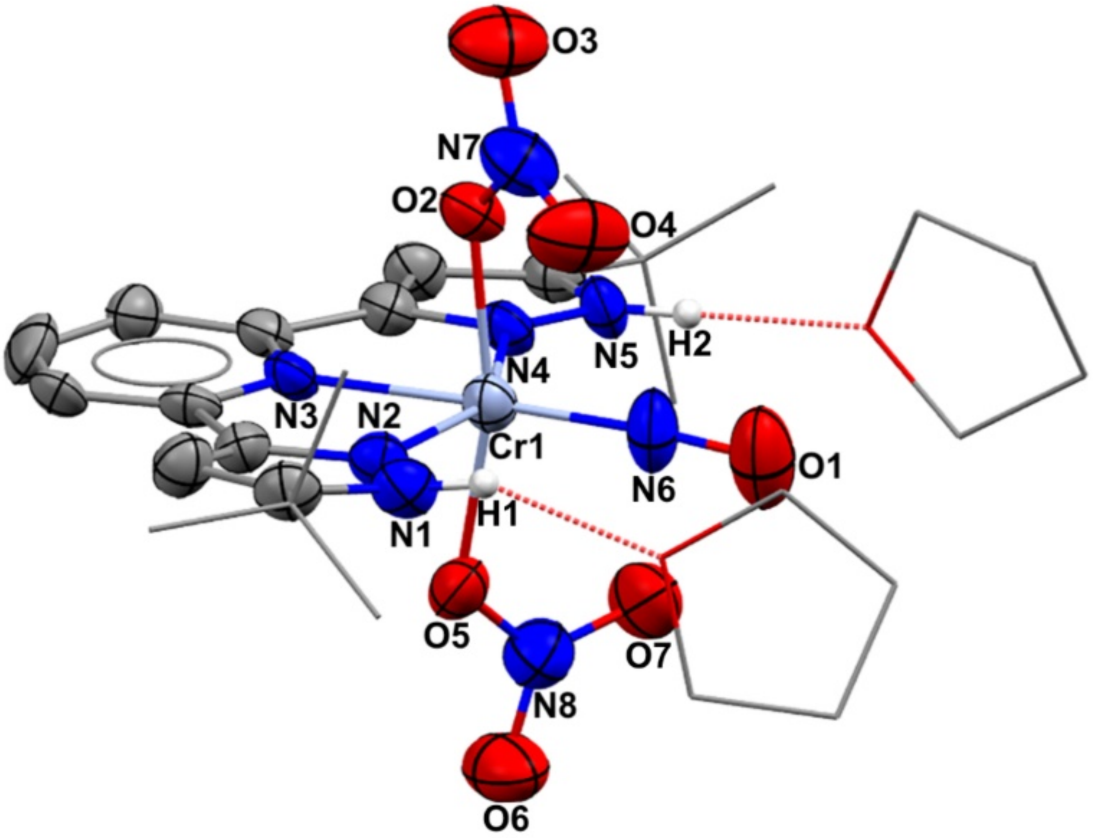


**Figure S11.** Mercury drawing of $(H_2L)Cr(NO_3)_2(NO)$, showing two THF molecules hydrogen bonding to two pyrazole NH protons.

| | $H_2LCr(NO_3)_3$ | $H_2LCr(NO_3)_2(NO)$ |
|---|---|---|
| Cr-$N_{pyd}$ | 2.026 | 2.078 |
| Cr-$N_{pyz}$ | 2.054/2.041 | 2.073/2.074 |
| $N_{pyz}$-$N_{pyz}$ | 1.345, 1.361 | 1.345,1.352 |
| Cr-O/$N_{equatorialNO3/NO}$ | 1.988 | 1.725 |
| N-$O_{NO3/NO}$ | 1.289/1.216/1.231 | 1.146 |
| Cr-$O_{axialNO3}$ | 1.980/1.985 | 2.017/2.002 |
| N-$O_{NO3}$ | 1.309/1.218/1.238/ | 1.298/1.219/1.251 |
| N-$O_{NO3}$ | 1.282/1.225/1.232 | 1.295/1.233/1.248 |
| <NNC | 111.8/110.8 | 112.4/110.9 |

**Table S1.** Comparison of bond lengths (Å) in related species. All esd's not shown are < 0.009 Å.

## Computational details

**General.** DFT[1] calculations were carried out using Gaussian 16.[2] Geometry optimizations were performed at the BP86/6-31G(d) level of theory.[3] Using either B3LYP[4] or BHHLYP[5] functionals, with 20 and 50% exchange contributions, gave results which consistently overstabilized the S = 3/2 state, while BP86 satisfied our benchmark criterion that the final product is more stable than any nitrato and nitrito precursor. We therefore completed the study with BP86, but we note that for $(H_2L)Cr(NO_3)_2(NO)$, both structure and vibrational frequencies were adequately modelled by all functionals; energy is much more sensitive to functional choice. Reoptimization of all species at the BP86/6-311G(d) level of theory did not change any conclusions, and thus analysis focused on the double zeta calculations. All optimized structures were confirmed to be minima by analyzing the harmonic frequencies.[6,7] Cartesian coordinates and thermodynamic data are summarized in Tables S?-S?.

### References.


1. Parr, R.G.; Yang, W. Density-functional theory of atoms and molecules; Oxford University Press: New York, 1989.
2. Gaussian 16, Revision B.01, M. J. Frisch, G. W. Trucks, H. B. Schlegel, G. E. Scuseria, M. A. Robb, J. R. Cheeseman, G. Scalmani, V. Barone, G. A. Petersson, H. Nakatsuji, X. Li, M. Caricato, A. V. Marenich, J. Bloino, B. G. Janesko, R. Gomperts, B. Mennucci, H. P. Hratchian, J. V. Ortiz, A. F. Izmaylov, J. L. Sonnenberg, D. Williams-Young, F. Ding, F. Lipparini, F. Egidi, J. Goings, B. Peng, A. Petrone, T. Henderson, D. Ranasinghe, V. G. Zakrzewski, J. Gao, N. Rega, G. Zheng, W. Liang, M. Hada, M. Ehara, K. Toyota, R. Fukuda, J. Hasegawa, M. Ishida, T. Nakajima, Y. Honda, O. Kitao, H. Nakai, T. Vreven, K. Throssell, J. A. Montgomery, Jr., J. E. Peralta, F. Ogliaro, M. J. Bearpark, J. J. Heyd, E. N. Brothers, K. N. Kudin, V. N. Staroverov, T. A. Keith, R. Kobayashi, J. Normand, K. Raghavachari, A. P. Rendell, J. C. Burant, S. S. Iyengar, J. Tomasi, M. Cossi, J. M. Millam, M. Klene, C. Adamo, R. Cammi, J. W. Ochterski, R. L. Martin, K. Morokuma, O. Farkas, J. B. Foresman, and D. J. Fox, Gaussian, Inc., Wallingford CT, 2016.
3. (a) Becke, A.D. *Phys. Rev. A.,* **1988**, *38*, 3098 (b) J. P. Perdew, *Phys. Rev. B*, **1986**, *33,* 8822.
4. (a) Vosko, S. H.; Wilk, L.; Nusair, M. *Can. J. Phys.* **1980**, *58*, 1200. (b) Lee, C.; Yang, W.; Parr, R.G. *Phys. Rev. B* **1988**, *37*, 785. (c) Becke, A. D. *J. Chem. Phys.* **1993**, *98*, 5648. (d) Stephens, P.J.; Devlin, F. J.; Chabalowski, C. F.; Frisch, M.J. *J. Phys. Chem.* **1994**, *98*, 11623.
5. Becke, A.D. *J. Chem. Phys.,* **1993**, *98*, 1372.
6. (a) Schlegel, H. B.; McDouall, J. J. in Computational Advances in Organic Chemistry; Oeretir, C; Csizmadia, I. G., Eds.; Kluwer Academic: Amsterdam, The Netherlands, 1991. (b) Bauernschmitt, R.; Ahlrichs, R. *J. Chem. Phys.* **1996**, *104*, 9047.
7. Schlegel, H. B. *WIREs Comput. Mol. Sci.* **2011**, *1*, 790.

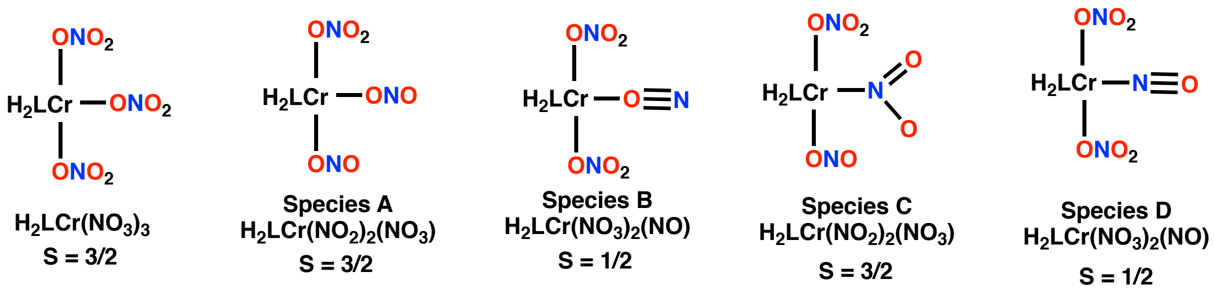


**Figure S12**: Labeling scheme for all calculated species. All subsequent figures and graphs use this labeling scheme

**Isonitrosyl analysis.** The isonitrosyl structure has much higher energy, but is interesting for its difference from the nitrosyl isomer. Its CrNO unit is bent (142°) and the NO bond is 0.06 Å longer, consistent with a double bond, and the Cr/O bond is longer (by 0.15 Å) than the Cr/N bond in the nitrosyl. Overall this suggests a Lewis structure (below) where the moderate bending at O is caused by a lone pair of $NO^{-1}$. A corresponding orbital representation (Figure S?) of the relevant orbitals shows much more occupancy of two orthogonal $\pi^*_{NO}$ orbitals. Together with the lower overlap value $S\alpha\beta$ which shows that these do not represent double occupancy of a single spatial orbital, but different orbital character, hence charge migration. This chromium has a higher oxidation state (by 0.5 e – See Table S?) than in the nitrosyl: isonitrosyl is more oxidizing towards this metal than is the nitrosyl. Judging by the corresponding orbital picture, $d^3$ Cr(III) is indicated.

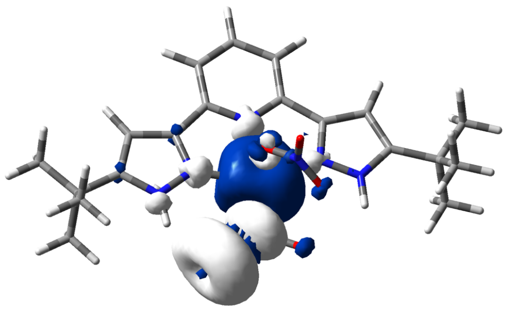

**Figure S13.** Spin density plot for species B plotted on an isosurface value of 0.002 a.u.

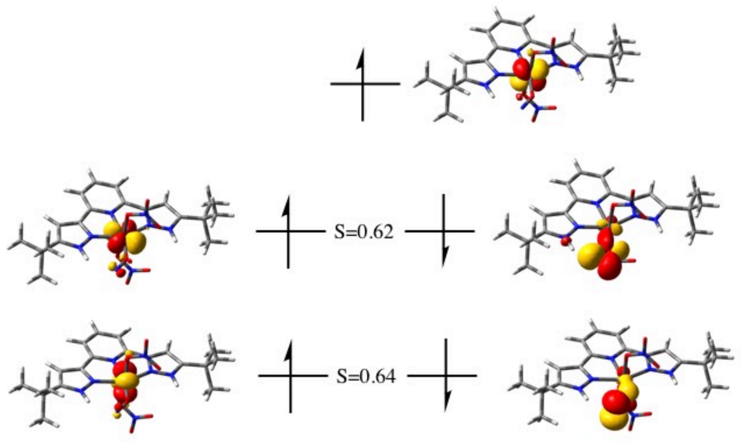


**Figure S14.** Corresponding orbital analysis of isonitrosyl species B

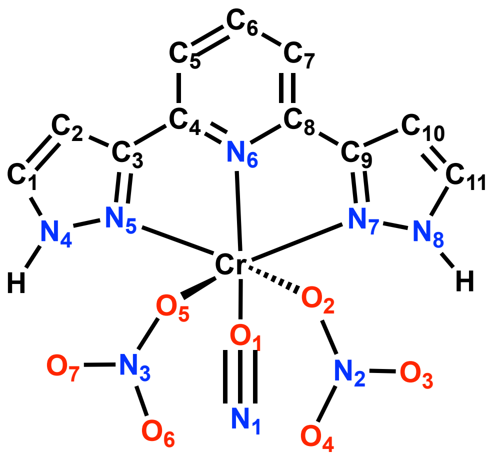


| | | | |
|---|---|---|---|
| **Cr** | 2.763 | **O6** | 0.009 |
| **N1** | –1.408 | **O7** | 0.003 |
| **N2** | 0.004 | **C1** | 0.010 |
| **N3** | 0.004 | **C2** | 0.000 |
| **N4** | 0.004 | **C3** | 0.009 |
| **N5** | –0.024 | **C4** | 0.011 |
| **N6** | –0.041 | **C5** | 0.001 |
| **N7** | –0.036 | **C6** | 0.007 |
| **N8** | –0.027 | **C7** | 0.001 |
| **O1** | –0.303 | **C8** | 0.008 |
| **O2** | –0.019 | **C9** | 0.016 |
| **O3** | 0.003 | **C10** | –0.002 |
| **O4** | 0.009 | **C11** | 0.016 |
| **O5** | –0.019 | | |

**Table S2.** Mulliken spin densities for species B.

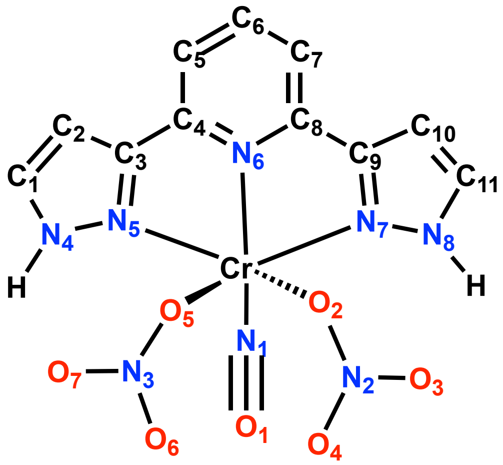


| | | | |
|---|---|---|---|
| **Cr** | 2.208 | **O6** | 0.002 |
| **N1** | –0.694 | **O7** | 0.005 |
| **N2** | 0.004 | **C1** | 0.012 |
| **N3** | –0.001 | **C2** | –0.003 |
| **N4** | 0.001 | **C3** | 0.013 |
| **N5** | –0.028 | **C4** | 0.005 |
| **N6** | –0.027 | **C5** | 0.004 |
| **N7** | –0.033 | **C6** | 0.004 |
| **N8** | 0.002 | **C7** | 0.004 |
| **O1** | –0.501 | **C8** | 0.005 |
| **O2** | –0.017 | **C9** | 0.017 |
| **O3** | 0.002 | **C10** | –0.003 |
| **O4** | 0.004 | **C11** | 0.016 |
| **O5** | –0.002 | | |

**Table S3.** Mulliken spin densities for species D. The expectation value of $S^2$ for this state D is 1.17, (vs. 0.75 for pure doublet).

To provide a second test of reliability of BP86, the NO stretching frequency in the computed vibrational spectrum was compared to the experimental. The results are summarized below in Table S4 including scaling factor.

| **Level of theory** | BP86/6-31G(d) |
|---|---|
| **Raw data ($cm^{-1}$)** | 1762.0 |
| **Scaling factor[1]** | .991 |
| **Scaled value ($cm^{-1}$)** | 1746.0 |

(1) *Phys. Rev. A.*, **1988**, *38*, 3098

**Table S4.** NO stretching frequency, raw and scaled data.

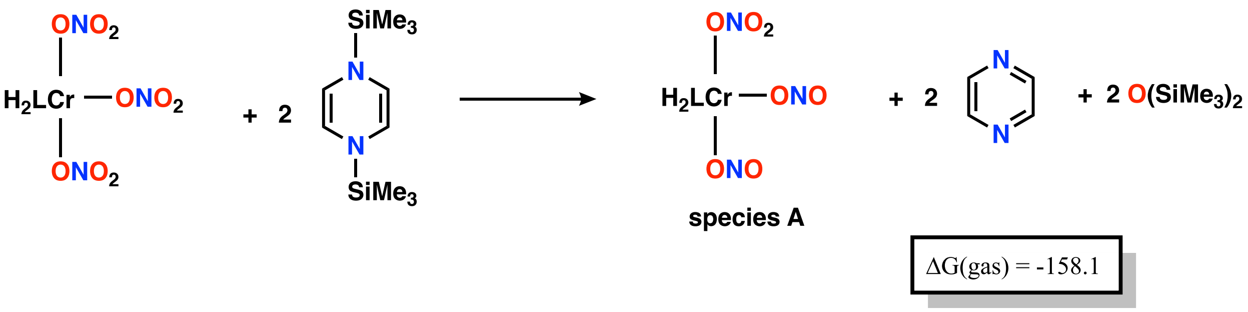


**Figure S15**. Overall reaction scheme for double deoxygenation of trisnitrate species, and free energy for transformation

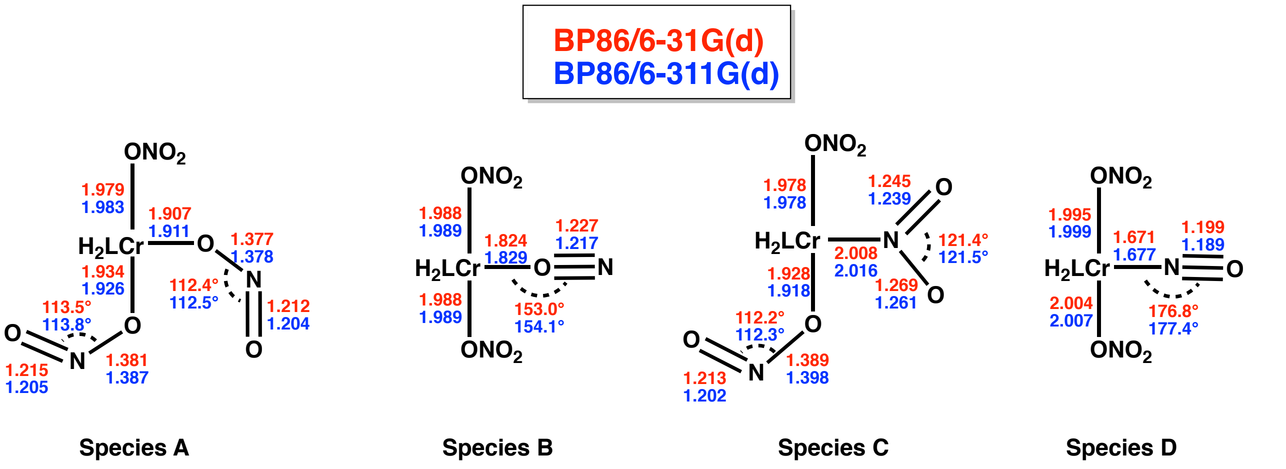


**Figure S16**. Bond length comparison between different levels of theory for Species A-D

**Table S5a**. Cartesian coordinates (in Å) for all optimized species at the BP86/6-31G(d) level of theory.

| | $H_2LCr(NO3)3$ | | | | Species A | | |
|---|---|---|---|---|---|---|---|
| Cr | -0.126225 | -0.348187 | 0.005778 | Cr | 0.118576 | -0.387491 | 0.099940 |
| N | 1.833632 | 0.187078 | 0.010682 | N | -1.794529 | 0.198657 | 0.036543 |
| N | 2.989234 | -0.477611 | -0.097531 | N | -2.959147 | -0.450607 | 0.116716 |
| N | -0.178713 | 1.695455 | 0.243453 | N | 0.250951 | 1.657838 | -0.107697 |
| N | -3.243858 | -0.648383 | 0.014155 | N | 3.293693 | -0.753624 | 0.168656 |
| N | -2.118689 | 0.075374 | 0.104081 | N | 2.165294 | -0.027669 | 0.096222 |
| C | 4.074618 | 0.345828 | 0.022674 | C | -4.027986 | 0.400492 | 0.054657 |
| C | 3.556760 | 1.636434 | 0.218959 | C | -3.486970 | 1.691008 | -0.071021 |
| H | 4.133025 | 2.550145 | 0.345855 | H | -4.047203 | 2.620392 | -0.145685 |
| C | 2.150651 | 1.494566 | 0.204318 | C | -2.082360 | 1.523617 | -0.077434 |
| C | 0.992991 | 2.376851 | 0.344145 | C | -0.899321 | 2.382143 | -0.164509 |
| C | 0.987160 | 3.764147 | 0.565618 | C | -0.847958 | 3.781745 | -0.286656 |
| H | 1.928407 | 4.314700 | 0.645209 | H | -1.769757 | 4.368084 | -0.334268 |
| C | -0.248495 | 4.419433 | 0.676684 | C | 0.410401 | 4.401277 | -0.344029 |
| H | -0.275914 | 5.501027 | 0.843867 | H | 0.474373 | 5.490040 | -0.439871 |
| C | -1.450645 | 3.699877 | 0.581819 | C | 1.588538 | 3.639270 | -0.275533 |
| H | -2.418735 | 4.199395 | 0.675736 | H | 2.572248 | 4.115366 | -0.313460 |
| C | -1.381867 | 2.313692 | 0.368545 | C | 1.473764 | 2.244386 | -0.152580 |
| C | -2.498735 | 1.367962 | 0.260132 | C | 2.560404 | 1.264607 | -0.048774 |
| C | -3.912137 | 1.442982 | 0.270054 | C | 3.974353 | 1.332643 | -0.061519 |
| H | -4.530294 | 2.331597 | 0.375605 | H | 4.595482 | 2.219771 | -0.162345 |
| C | -4.371110 | 0.127844 | 0.108619 | C | 4.426840 | 0.015529 | 0.079776 |
| C | 5.497324 | -0.180832 | -0.048219 | C | -5.460628 | -0.100850 | 0.103487 |
| C | 6.485813 | 1.003645 | 0.016827 | C | -6.425097 | 1.104672 | 0.133994 |
| H | 6.382018 | 1.566011 | 0.962105 | H | -6.320021 | 1.730310 | -0.770550 |
| H | 6.335749 | 1.702671 | -0.825254 | H | -6.251819 | 1.739459 | 1.021369 |
| H | 7.521827 | 0.626185 | -0.036771 | H | -7.468091 | 0.744992 | 0.173229 |
| C | 5.750824 | -1.132096 | 1.152605 | C | -5.744390 | -0.958691 | -1.159277 |
| H | 5.617063 | -0.605143 | 2.113389 | H | -5.607712 | -0.367408 | -2.081372 |
| H | 6.784419 | -1.520193 | 1.110875 | H | -6.785430 | -1.328109 | -1.134426 |
| H | 5.064182 | -1.996762 | 1.144681 | H | -5.074423 | -1.834115 | -1.219205 |
| C | 5.701138 | -0.950617 | -1.379967 | C | -5.668477 | -0.959824 | 1.378775 |
| H | 6.740211 | -1.320398 | -1.441660 | H | -6.714906 | -1.310759 | 1.425219 |
| H | 5.512842 | -0.299431 | -2.251278 | H | -5.456657 | -0.378156 | 2.292762 |
| H | 5.031675 | -1.825597 | -1.458568 | H | -5.018131 | -1.852476 | 1.386695 |
| C | -5.768148 | -0.463497 | 0.034534 | C | 5.820354 | -0.585995 | 0.139065 |
| C | -6.809516 | 0.669219 | 0.166028 | C | 6.867944 | 0.540761 | 0.005238 |

| | | | | | | | |
|---|---|---|---|---|---|---|---|
| H | -6.714076 | 1.196628 | 1.131873 | H | 6.766680 | 1.074523 | -0.956443 |
| H | -7.827377 | 0.245574 | 0.112575 | H | 7.883770 | 0.110800 | 0.047193 |
| H | -6.708579 | 1.408157 | -0.648737 | H | 6.778317 | 1.275505 | 0.825115 |
| C | -5.962637 | -1.479240 | 1.192186 | C | 6.000194 | -1.596118 | -1.025760 |
| H | -5.253056 | -2.323519 | 1.125617 | H | 5.288050 | -2.438330 | -0.957961 |
| H | -6.983319 | -1.899539 | 1.153133 | H | 7.019364 | -2.021180 | -0.998827 |
| H | -5.824173 | -0.996673 | 2.175250 | H | 5.853833 | -1.107738 | -2.004697 |
| C | -5.955639 | -1.179944 | -1.329749 | C | 6.015909 | -1.310978 | 1.497651 |
| H | -5.812210 | -0.480915 | -2.171863 | H | 5.883621 | -0.615536 | 2.344573 |
| H | -6.976206 | -1.597153 | -1.395595 | H | 7.034412 | -1.734927 | 1.552825 |
| H | -5.245982 | -2.016801 | -1.459301 | H | 5.302253 | -2.144332 | 1.629031 |
| H | -3.142085 | -1.657232 | -0.114156 | H | 3.206976 | -1.764252 | 0.275703 |
| H | 2.897144 | -1.494070 | -0.223134 | H | -2.873820 | -1.477882 | 0.204630 |
| O | -0.319955 | -0.420660 | -1.943107 | O | 0.205351 | -0.090029 | 2.008962 |
| O | -0.184652 | -0.388604 | 1.977636 | O | 0.374443 | -0.526808 | -1.857473 |
| O | -0.555761 | -2.252520 | -0.062054 | O | 0.003270 | -2.279310 | 0.313238 |
| N | 0.645661 | -1.225734 | 2.656972 | N | -0.478919 | -1.178930 | -2.690451 |
| N | 0.331883 | -3.117100 | -0.605969 | N | -1.244862 | -2.844561 | 0.453320 |
| N | 0.110324 | 0.478357 | -2.870014 | N | -0.846322 | -0.537205 | 2.783983 |
| O | 0.512776 | -1.239077 | 3.882810 | O | -0.096677 | -1.301274 | -3.858961 |
| O | 1.466080 | -1.897852 | 2.001227 | O | -1.563457 | -1.588415 | -2.233853 |
| O | 1.455235 | -2.672404 | -0.937116 | O | -1.216507 | -4.046185 | 0.610996 |
| O | -0.040765 | -4.280614 | -0.736901 | O | -0.680605 | -0.307945 | 3.965572 |
| O | -0.150029 | 0.192806 | -4.041961 | | | | |
| O | 0.714122 | 1.498706 | -2.487260 | | | | |
| | **Species B** | | | | **Species C** | | |
| Cr | 0.126317 | -0.420121 | 0.000010 | Cr | 0.112385 | -0.383435 | 0.110038 |
| N | -1.875577 | 0.064188 | -0.000008 | N | -1.863440 | 0.095767 | 0.021820 |
| N | -3.038521 | -0.602248 | -0.000010 | N | -3.005482 | -0.595279 | 0.112711 |
| N | 0.128189 | 1.614342 | -0.000013 | N | 0.110277 | 1.679383 | -0.165479 |
| N | 3.227110 | -0.660982 | 0.000017 | N | 3.177726 | -0.626714 | 0.135910 |
| N | 2.092177 | 0.056203 | 0.000011 | N | 2.062208 | 0.104868 | 0.056007 |
| C | -4.126580 | 0.227849 | -0.000019 | C | -4.111196 | 0.208005 | 0.047894 |
| C | -3.608993 | 1.530407 | -0.000036 | C | -3.622202 | 1.516208 | -0.091800 |
| H | -4.183654 | 2.453846 | -0.000052 | H | -4.219551 | 2.421476 | -0.173804 |
| C | -2.200987 | 1.384957 | -0.000022 | C | -2.211717 | 1.405799 | -0.104548 |
| C | -1.055434 | 2.289151 | -0.000024 | C | -1.078039 | 2.331182 | -0.216919 |
| C | -1.080631 | 3.694908 | -0.000037 | C | -1.110449 | 3.729890 | -0.361208 |
| H | -2.036145 | 4.226714 | -0.000047 | H | -2.063534 | 4.264213 | -0.404878 |
| C | 0.137042 | 4.389788 | -0.000039 | C | 0.111368 | 4.416682 | -0.445988 |

| | | | | | | | |
|---|---|---|---|---|---|---|---|
| H | 0.140720 | 5.484443 | -0.000049 | H | 0.111966 | 5.505710 | -0.559730 |
| C | 1.353231 | 3.686274 | -0.000028 | C | 1.333405 | 3.726318 | -0.382021 |
| H | 2.311799 | 4.212477 | -0.000029 | H | 2.286649 | 4.258891 | -0.440858 |
| C | 1.317806 | 2.284152 | -0.000015 | C | 1.302147 | 2.328930 | -0.234367 |
| C | 2.454652 | 1.363873 | -0.000004 | C | 2.433136 | 1.398558 | -0.123920 |
| C | 3.865842 | 1.458959 | -0.000007 | C | 3.846667 | 1.466670 | -0.150603 |
| H | 4.472881 | 2.361468 | -0.000017 | H | 4.468480 | 2.350233 | -0.277848 |
| C | 4.339910 | 0.137887 | 0.000004 | C | 4.301949 | 0.147593 | 0.018601 |
| C | -5.548341 | -0.306552 | -0.000012 | C | -5.521343 | -0.353648 | 0.096933 |
| C | -6.540056 | 0.876914 | -0.000059 | C | -6.534687 | 0.810286 | 0.150911 |
| H | -6.414217 | 1.509914 | -0.896612 | H | -6.461363 | 1.452858 | -0.744768 |
| H | -6.414234 | 1.509970 | 0.896458 | H | -6.382011 | 1.438408 | 1.046605 |
| H | -7.575553 | 0.494248 | -0.000056 | H | -7.561554 | 0.407020 | 0.190258 |
| C | -5.775937 | -1.169278 | -1.270227 | C | -5.775486 | -1.202173 | -1.178772 |
| H | -5.619296 | -0.576741 | -2.188315 | H | -5.675959 | -0.588609 | -2.091078 |
| H | -6.810616 | -1.556283 | -1.279266 | H | -6.797414 | -1.621206 | -1.152423 |
| H | -5.093376 | -2.036670 | -1.309755 | H | -5.065409 | -2.043809 | -1.261011 |
| C | -5.775955 | -1.169194 | 1.270257 | C | -5.687566 | -1.240049 | 1.359376 |
| H | -6.810633 | -1.556203 | 1.279306 | H | -6.720101 | -1.629369 | 1.407533 |
| H | -5.619330 | -0.576595 | 2.188308 | H | -5.489129 | -0.666623 | 2.281360 |
| H | -5.093390 | -2.036580 | 1.309853 | H | -5.007040 | -2.110207 | 1.348582 |
| C | 5.744894 | -0.439269 | 0.000008 | C | 5.698851 | -0.445190 | 0.079132 |
| C | 6.772452 | 0.713207 | -0.000022 | C | 6.742487 | 0.680633 | -0.086939 |
| H | 6.666221 | 1.349855 | -0.896486 | H | 6.633127 | 1.193740 | -1.059150 |
| H | 7.795639 | 0.298892 | -0.000020 | H | 7.759795 | 0.254127 | -0.043594 |
| H | 6.666233 | 1.349892 | 0.896417 | H | 6.657820 | 1.432959 | 0.717619 |
| C | 5.946261 | -1.309889 | -1.269303 | C | 5.869816 | -1.480748 | -1.064243 |
| H | 5.245006 | -2.162846 | -1.300134 | H | 5.152959 | -2.315868 | -0.972301 |
| H | 6.971718 | -1.720518 | -1.282646 | H | 6.888123 | -1.908067 | -1.033403 |
| H | 5.799308 | -0.716144 | -2.188306 | H | 5.720651 | -1.012424 | -2.052784 |
| C | 5.946275 | -1.309834 | 1.269355 | C | 5.904497 | -1.141859 | 1.450785 |
| H | 5.799334 | -0.716050 | 2.188334 | H | 5.781515 | -0.427752 | 2.283715 |
| H | 6.971731 | -1.720464 | 1.282704 | H | 6.922849 | -1.566598 | 1.506222 |
| H | 5.245018 | -2.162788 | 1.300231 | H | 5.187749 | -1.968197 | 1.603050 |
| H | 3.124420 | -1.690215 | 0.000023 | H | 2.987686 | -1.642360 | 0.274167 |
| H | -2.985629 | -1.621011 | -0.000003 | H | -2.914573 | -1.611700 | 0.187238 |
| O | 0.143701 | -0.288248 | 1.983884 | O | 0.156157 | -0.069660 | 2.011624 |
| O | 0.143720 | -0.288296 | -1.983868 | O | 0.277590 | -0.619725 | -1.844587 |
| N | -0.644133 | -1.149054 | -2.669369 | N | -0.607949 | -1.377722 | -2.542970 |
| N | -0.644135 | -1.149011 | 2.669400 | N | -0.967042 | -0.381424 | 2.767516 |

| | | | | | | | |
|---|---|---|---|---|---|---|---|
| O | -0.596618 | -1.079769 | -3.900960 | O | -0.477879 | -1.374888 | -3.769400 |
| O | -1.365378 | -1.935687 | -2.012729 | O | -1.470004 | -2.014268 | -1.901786 |
| O | -0.596591 | -1.079733 | 3.900990 | O | -0.813974 | -0.125711 | 3.942861 |
| O | -1.365271 | -1.935755 | 2.012774 | O | -0.627437 | -2.981096 | 0.807126 |
| O | 0.656304 | -2.165805 | 0.000029 | O | 1.539042 | -2.869766 | 0.503812 |
| N | 1.506712 | -3.050471 | 0.000019 | N | 0.391311 | -2.333453 | 0.501531 |

**Species D**

| | | | |
|---|---|---|---|
| Cr | 0.068256 | -0.513795 | 0.367504 |
| N | -1.884869 | -0.023769 | 0.190033 |
| N | -3.049993 | -0.625873 | 0.490371 |
| N | 0.104791 | 1.288612 | -0.650687 |
| N | 3.200657 | -0.841558 | 0.264389 |
| N | 2.049501 | -0.203239 | -0.020280 |
| C | -4.137027 | 0.091045 | 0.061264 |
| C | -3.618621 | 1.251152 | -0.527656 |
| H | -4.192001 | 2.065435 | -0.964876 |
| C | -2.212008 | 1.137774 | -0.437403 |
| C | -1.069667 | 1.911436 | -0.921251 |
| C | -1.086537 | 3.125448 | -1.628008 |
| H | -2.032757 | 3.629013 | -1.843764 |
| C | 0.137943 | 3.673863 | -2.040538 |
| H | 0.150926 | 4.622506 | -2.587046 |
| C | 1.345736 | 3.014102 | -1.764023 |
| H | 2.304503 | 3.427820 | -2.088553 |
| C | 1.291909 | 1.799512 | -1.058461 |
| C | 2.409912 | 0.936492 | -0.669983 |
| C | 3.818275 | 0.996590 | -0.788783 |
| H | 4.411771 | 1.780263 | -1.254133 |
| C | 4.308591 | -0.161117 | -0.173792 |
| C | -5.556311 | -0.422326 | 0.228979 |
| C | -6.553126 | 0.681561 | -0.186206 |
| H | -6.421837 | 0.966959 | -1.245234 |
| H | -6.436678 | 1.586059 | 0.436922 |
| H | -7.587214 | 0.314561 | -0.064124 |
| C | -5.757138 | -1.666630 | -0.679112 |
| H | -5.607622 | -1.409869 | -1.742206 |
| H | -6.782873 | -2.059087 | -0.557687 |
| H | -5.051433 | -2.479011 | -0.430258 |
| C | -5.804653 | -0.807674 | 1.710858 |

| | | | |
|---|---|---|---|
| H | -6.843677 | -1.161069 | 1.836274 |
| H | -5.648627 | 0.055938 | 2.380499 |
| H | -5.136144 | -1.621565 | 2.044773 |
| C | 5.717860 | -0.685406 | 0.040750 |
| C | 6.730482 | 0.315209 | -0.557668 |
| H | 6.576300 | 0.445298 | -1.643653 |
| H | 7.757753 | -0.058985 | -0.404960 |
| H | 6.655402 | 1.304407 | -0.072207 |
| C | 5.875138 | -2.059275 | -0.664368 |
| H | 5.185661 | -2.817838 | -0.251491 |
| H | 6.904223 | -2.436379 | -0.526320 |
| H | 5.678563 | -1.977327 | -1.747477 |
| C | 5.987949 | -0.841572 | 1.561402 |
| H | 5.867863 | 0.120115 | 2.089472 |
| H | 7.019784 | -1.200973 | 1.723927 |
| H | 5.305018 | -1.572548 | 2.031129 |
| H | 3.149928 | -1.717706 | 0.782023 |
| H | -2.996893 | -1.605794 | 0.771100 |
| O | 0.419805 | 0.410335 | 2.111085 |
| O | -0.046994 | -1.270346 | -1.474912 |
| N | -0.862615 | -2.305273 | -1.779796 |
| N | 0.189375 | 1.701122 | 2.442196 |
| O | -0.785138 | -2.728099 | -2.939675 |
| O | -1.636768 | -2.754555 | -0.903095 |
| O | 0.923445 | 2.166222 | 3.325709 |
| O | -0.742897 | 2.319668 | 1.888224 |
| N | 0.088959 | -1.968012 | 1.190792 |
| O | 0.157175 | -2.988767 | 1.815496 |

**Table S5b.** Cartesian coordinates (in Å) for all optimized species at the BP86/6-311G(d) level of theory.

| | $H_2LCr(NO_3)_3$ | | | | Species A | | |
|---|---|---|---|---|---|---|---|
| Cr | -0.128459 | -0.348441 | -0.019965 | Cr | 0.120821 | -0.384186 | 0.135395 |
| N | 1.827353 | 0.189828 | -0.017997 | N | -1.788261 | 0.208332 | 0.087157 |
| N | 2.981787 | -0.469092 | 0.106541 | N | -2.951061 | -0.441860 | 0.139234 |
| N | -0.191061 | 1.689111 | -0.262969 | N | 0.262545 | 1.661047 | -0.037340 |
| N | -3.250960 | -0.658365 | -0.007765 | N | 3.299923 | -0.766159 | 0.138777 |
| N | -2.129265 | 0.064911 | -0.108999 | N | 2.174900 | -0.036215 | 0.103770 |
| C | 4.062051 | 0.357650 | -0.013548 | C | -4.015690 | 0.409978 | 0.062998 |
| C | 3.541103 | 1.642636 | -0.223726 | C | -3.472920 | 1.700220 | -0.033949 |

| | | | | | | | |
|---|---|---|---|---|---|---|---|
| H | 4.113132 | 2.557228 | -0.350941 | H | -4.030230 | 2.629793 | -0.107263 |
| C | 2.137244 | 1.495095 | -0.218016 | C | -2.070007 | 1.532280 | -0.016122 |
| C | 0.977394 | 2.371249 | -0.365307 | C | -0.884534 | 2.387324 | -0.086252 |
| C | 0.966113 | 3.755290 | -0.588779 | C | -0.827492 | 3.784938 | -0.194436 |
| H | 1.903313 | 4.310147 | -0.668378 | H | -1.745088 | 4.375976 | -0.234927 |
| C | -0.269216 | 4.404916 | -0.700142 | C | 0.430600 | 4.399272 | -0.249591 |
| H | -0.300132 | 5.484857 | -0.868785 | H | 0.498112 | 5.487394 | -0.334119 |
| C | -1.466934 | 3.683594 | -0.602103 | C | 1.603853 | 3.633654 | -0.196933 |
| H | -2.434431 | 4.181685 | -0.694884 | H | 2.586908 | 4.107798 | -0.238830 |
| C | -1.394712 | 2.300978 | -0.386013 | C | 1.485663 | 2.240482 | -0.088542 |
| C | -2.508532 | 1.353916 | -0.269481 | C | 2.568999 | 1.255663 | -0.016912 |
| C | -3.920707 | 1.428756 | -0.269898 | C | 3.981577 | 1.320578 | -0.052745 |
| H | -4.538623 | 2.315670 | -0.376022 | H | 4.602331 | 2.207259 | -0.143369 |
| C | -4.377603 | 0.116644 | -0.099048 | C | 4.432101 | 0.001820 | 0.048111 |
| C | 5.484989 | -0.162059 | 0.064155 | C | -5.447490 | -0.090352 | 0.057610 |
| C | 6.468647 | 1.024418 | 0.022230 | C | -6.412459 | 1.108136 | 0.157027 |
| H | 6.309754 | 1.714583 | 0.866758 | H | -6.304058 | 1.790760 | -0.701654 |
| H | 6.378171 | 1.595311 | -0.916381 | H | -6.251513 | 1.684408 | 1.082695 |
| H | 7.503627 | 0.652664 | 0.084146 | H | -7.453687 | 0.748834 | 0.162498 |
| C | 5.682557 | -0.946489 | 1.385191 | C | -5.710086 | -0.854875 | -1.265633 |
| H | 5.479514 | -0.312142 | 2.262522 | H | -5.557995 | -0.202182 | -2.139687 |
| H | 6.721872 | -1.306550 | 1.455412 | H | -6.748734 | -1.224143 | -1.287631 |
| H | 5.024917 | -1.828465 | 1.447395 | H | -5.039850 | -1.721220 | -1.379006 |
| C | 5.752141 | -1.094989 | -1.145079 | C | -5.677653 | -1.035436 | 1.262606 |
| H | 6.785044 | -1.478138 | -1.104117 | H | -6.724916 | -1.378662 | 1.274671 |
| H | 5.622149 | -0.559047 | -2.098599 | H | -5.471984 | -0.527035 | 2.217828 |
| H | 5.072318 | -1.961587 | -1.155570 | H | -5.040210 | -1.932952 | 1.214794 |
| C | -5.773516 | -0.471380 | -0.013067 | C | 5.824248 | -0.601272 | 0.066853 |
| C | -6.813942 | 0.659114 | -0.144075 | C | 6.870384 | 0.524088 | -0.063246 |
| H | -6.711684 | 1.401814 | 0.663681 | H | 6.755269 | 1.078485 | -1.008635 |
| H | -7.830036 | 0.238851 | -0.082288 | H | 7.884289 | 0.094129 | -0.050872 |
| H | -6.728301 | 1.181100 | -1.110918 | H | 6.802574 | 1.240564 | 0.771354 |
| C | -5.955226 | -1.178441 | 1.354019 | C | 5.981353 | -1.584394 | -1.120731 |
| H | -5.253789 | -2.018660 | 1.484684 | H | 5.278137 | -2.431033 | -1.056464 |
| H | -6.975355 | -1.587879 | 1.432811 | H | 6.999885 | -2.005273 | -1.129351 |
| H | -5.800958 | -0.479303 | 2.190879 | H | 5.810399 | -1.079486 | -2.084348 |
| C | -5.978149 | -1.491168 | -1.162002 | C | 6.047077 | -1.353852 | 1.403133 |
| H | -5.840288 | -1.018326 | -2.147110 | H | 5.928872 | -0.680543 | 2.266623 |
| H | -6.998654 | -1.905113 | -1.120095 | H | 7.064971 | -1.775338 | 1.433397 |
| H | -5.277187 | -2.339252 | -1.094947 | H | 5.341562 | -2.191199 | 1.531393 |

| | | | |
|---|---|---|---|
| H | -3.151127 | -1.664441 | 0.124889 |
| H | 2.893970 | -1.482869 | 0.245676 |
| O | -0.179207 | -0.432621 | -1.986494 |
| O | -0.285403 | -0.381371 | 1.932217 |
| O | -0.490382 | -2.264966 | 0.080664 |
| N | 0.120075 | 0.525704 | 2.861494 |
| N | 0.381235 | -3.133460 | 0.644407 |
| N | 0.651149 | -1.250904 | -2.690487 |
| O | 0.693873 | 1.558139 | 2.481145 |
| O | -0.130907 | 0.231145 | 4.025968 |
| O | 1.519132 | -2.713149 | 0.934067 |
| O | -0.021713 | -4.272711 | 0.823425 |
| O | 0.469748 | -1.278127 | -3.903009 |
| O | 1.512535 | -1.887845 | -2.064501 |

**Species B**

| | | | |
|---|---|---|---|
| Cr | 0.125571 | -0.410251 | 0.000013 |
| N | -1.871730 | 0.069337 | -0.000005 |
| N | -3.031910 | -0.596176 | -0.000008 |
| N | 0.134546 | 1.615503 | -0.000018 |
| N | 3.230638 | -0.666799 | 0.000014 |
| N | 2.097264 | 0.050211 | 0.000007 |
| C | -4.117059 | 0.234163 | -0.000023 |
| C | -3.599380 | 1.534518 | -0.000034 |
| H | -4.172602 | 2.457142 | -0.000047 |
| C | -2.192869 | 1.388381 | -0.000024 |
| C | -1.046811 | 2.290551 | -0.000029 |
| C | -1.066545 | 3.693411 | -0.000046 |
| H | -2.018000 | 4.229922 | -0.000055 |
| C | 0.151261 | 4.383210 | -0.000052 |
| H | 0.158072 | 5.476499 | -0.000066 |
| C | 1.363482 | 3.677809 | -0.000042 |
| H | 2.321053 | 4.203270 | -0.000047 |
| C | 1.325322 | 2.278644 | -0.000025 |
| C | 2.458723 | 1.355204 | -0.000013 |
| C | 3.869080 | 1.449775 | -0.000019 |
| H | 4.475109 | 2.351161 | -0.000034 |
| C | 4.342624 | 0.131550 | -0.000001 |
| C | -5.537128 | -0.299312 | -0.000024 |
| C | -6.529620 | 0.880324 | -0.000049 |
| H | -6.408773 | 1.513322 | -0.894227 |

| | | | |
|---|---|---|---|
| H | 3.213515 | -1.777148 | 0.216045 |
| H | -2.863772 | -1.470983 | 0.198761 |
| O | 0.219003 | -0.156959 | 2.045288 |
| O | 0.330731 | -0.490691 | -1.833876 |
| O | -0.045828 | -2.281308 | 0.292114 |
| N | -0.529575 | -1.084186 | -2.697345 |
| N | -1.297972 | -2.842694 | 0.410502 |
| N | -0.805096 | -0.655523 | 2.837716 |
| O | -0.152971 | -1.147942 | -3.865832 |
| O | -1.614724 | -1.502987 | -2.263074 |
| O | -1.284156 | -4.040274 | 0.532104 |
| O | -0.606738 | -0.479395 | 4.013256 |

**Species C**

| | | | |
|---|---|---|---|
| Cr | 0.115550 | -0.380098 | 0.165411 |
| N | -1.861400 | 0.100672 | 0.072021 |
| N | -3.004057 | -0.586758 | 0.147428 |
| N | 0.117929 | 1.676900 | -0.145036 |
| N | 3.188729 | -0.628689 | 0.135009 |
| N | 2.073163 | 0.100695 | 0.072331 |
| C | -4.104303 | 0.216058 | 0.041591 |
| C | -3.611211 | 1.520145 | -0.098474 |
| H | -4.203635 | 2.424444 | -0.204179 |
| C | -2.202537 | 1.407160 | -0.078738 |
| C | -1.067796 | 2.327978 | -0.201172 |
| C | -1.097388 | 3.721389 | -0.368598 |
| H | -2.047554 | 4.258035 | -0.415408 |
| C | 0.122956 | 4.402466 | -0.474169 |
| H | 0.125148 | 5.488103 | -0.605931 |
| C | 1.341399 | 3.711371 | -0.410203 |
| H | 2.293272 | 4.241246 | -0.488899 |
| C | 1.308935 | 2.319409 | -0.238873 |
| C | 2.438835 | 1.389265 | -0.131727 |
| C | 3.850371 | 1.457511 | -0.191337 |
| H | 4.467482 | 2.338491 | -0.344205 |
| C | 4.308821 | 0.143136 | -0.017494 |
| C | -5.513802 | -0.344295 | 0.039536 |
| C | -6.527792 | 0.813174 | 0.138499 |
| H | -6.448820 | 1.498717 | -0.720987 |

| | | | | | | | |
|---|---|---|---|---|---|---|---|
| H | -6.408785 | 1.513349 | 0.894112 | H | -6.389773 | 1.396526 | 1.063278 |
| H | -7.562780 | 0.498461 | -0.000050 | H | -7.553119 | 0.410812 | 0.144804 |
| C | -5.764172 | -1.160210 | -1.268577 | C | -5.744019 | -1.119896 | -1.283769 |
| H | -5.607021 | -0.570100 | -2.185198 | H | -5.637126 | -0.456893 | -2.156872 |
| H | -6.796181 | -1.547261 | -1.281398 | H | -6.760236 | -1.547052 | -1.298994 |
| H | -5.084109 | -2.026062 | -1.311217 | H | -5.026862 | -1.946882 | -1.406543 |
| C | -5.764187 | -1.160170 | 1.268554 | C | -5.704312 | -1.296549 | 1.245652 |
| H | -6.796195 | -1.547224 | 1.281373 | H | -6.739487 | -1.673904 | 1.264399 |
| H | -5.607050 | -0.570030 | 2.185157 | H | -5.508330 | -0.782608 | 2.199752 |
| H | -5.084121 | -2.026018 | 1.311230 | H | -5.040304 | -2.174746 | 1.193658 |
| C | 5.746395 | -0.443897 | 0.000002 | C | 5.705257 | -0.448235 | 0.016957 |
| C | 6.773231 | 0.706061 | -0.000021 | C | 6.744373 | 0.671092 | -0.193173 |
| H | 6.671177 | 1.342388 | -0.894082 | H | 6.614796 | 1.168663 | -1.168103 |
| H | 7.794592 | 0.293881 | -0.000019 | H | 7.760372 | 0.246386 | -0.169175 |
| H | 6.671185 | 1.342417 | 0.894019 | H | 6.684846 | 1.435540 | 0.598668 |
| C | 5.949508 | -1.312693 | -1.267283 | C | 5.848289 | -1.500282 | -1.111788 |
| H | 5.256027 | -2.168742 | -1.298647 | H | 5.141334 | -2.336389 | -0.988372 |
| H | 6.974277 | -1.718145 | -1.285573 | H | 6.866108 | -1.923432 | -1.105635 |
| H | 5.797843 | -0.723263 | -2.185310 | H | 5.669314 | -1.051892 | -2.101852 |
| C | 5.949519 | -1.312651 | 1.267314 | C | 5.946551 | -1.121622 | 1.391662 |
| H | 5.797862 | -0.723191 | 2.185323 | H | 5.841862 | -0.397920 | 2.215515 |
| H | 6.974288 | -1.718103 | 1.285609 | H | 6.964739 | -1.542062 | 1.431900 |
| H | 5.256037 | -2.168699 | 1.298712 | H | 5.239667 | -1.946439 | 1.576229 |
| H | 3.138686 | -1.691376 | 0.000027 | H | 3.013756 | -1.640051 | 0.283774 |
| H | -2.981786 | -1.612761 | 0.000002 | H | -2.921777 | -1.603109 | 0.197469 |
| O | 0.139504 | -0.310666 | 1.986483 | O | 0.165683 | -0.064663 | 2.056815 |
| O | 0.139504 | -0.310730 | -1.986457 | O | 0.236599 | -0.674714 | -1.787241 |
| N | -0.651442 | -1.146370 | -2.697204 | N | -0.654826 | -1.399194 | -2.508599 |
| N | -0.651398 | -1.146318 | 2.697266 | N | -0.962325 | -0.313374 | 2.844483 |
| O | -0.565382 | -1.071784 | -3.920052 | O | -0.486163 | -1.406099 | -3.723862 |
| O | -1.408014 | -1.914518 | -2.069205 | O | -1.558273 | -1.997508 | -1.897393 |
| O | -0.565352 | -1.071664 | 3.920110 | O | -0.769700 | -0.073768 | 4.006521 |
| O | -1.407868 | -1.914595 | 2.069304 | O | -0.640256 | -3.000940 | 0.845573 |
| O | 0.617690 | -2.171715 | 0.000041 | O | 1.516466 | -2.861705 | 0.549044 |
| N | 1.423833 | -3.083307 | 0.000040 | N | 0.367148 | -2.341924 | 0.552921 |

**Species D**

| | | | |
|---|---|---|---|
| Cr | 0.074060 | -0.505318 | 0.376923 |
| N | -1.881632 | -0.010526 | 0.197345 |
| N | -3.046623 | -0.609879 | 0.496700 |

| | | | |
|---|---|---|---|
| N | 0.112557 | 1.278111 | -0.671732 |
| N | 3.212541 | -0.831579 | 0.279189 |
| N | 2.061049 | -0.199736 | -0.011298 |
| C | -4.128137 | 0.101378 | 0.049814 |
| C | -3.606479 | 1.253709 | -0.545529 |
| H | -4.175343 | 2.063307 | -0.993571 |
| C | -2.202047 | 1.140872 | -0.444479 |
| C | -1.058685 | 1.901911 | -0.941518 |
| C | -1.073005 | 3.106899 | -1.657516 |
| H | -2.015864 | 3.614845 | -1.871079 |
| C | 0.149084 | 3.644813 | -2.081174 |
| H | 0.163561 | 4.587381 | -2.635412 |
| C | 1.352868 | 2.984489 | -1.803276 |
| H | 2.309984 | 3.393227 | -2.134695 |
| C | 1.297987 | 1.779987 | -1.086107 |
| C | 2.415284 | 0.923455 | -0.686556 |
| C | 3.821486 | 0.981679 | -0.815382 |
| H | 4.410217 | 1.754390 | -1.301195 |
| C | 4.315618 | -0.160862 | -0.181229 |
| C | -5.547322 | -0.412761 | 0.200231 |
| C | -6.541013 | 0.695717 | -0.201637 |
| H | -6.411081 | 0.996151 | -1.253996 |
| H | -6.428807 | 1.590495 | 0.431755 |
| H | -7.574032 | 0.330301 | -0.088451 |
| C | -5.742479 | -1.638448 | -0.730245 |
| H | -5.584718 | -1.364604 | -1.785295 |
| H | -6.766979 | -2.032472 | -0.626731 |
| H | -5.041941 | -2.455455 | -0.494356 |
| C | -5.807936 | -0.823408 | 1.670273 |
| H | -6.847898 | -1.169780 | 1.785022 |
| H | -5.650125 | 0.022771 | 2.357451 |
| H | -5.152243 | -1.648059 | 1.994466 |
| C | 5.726213 | -0.677274 | 0.032537 |
| C | 6.731848 | 0.310699 | -0.592175 |
| H | 6.573796 | 0.419489 | -1.677356 |
| H | 7.759057 | -0.056660 | -0.440553 |
| H | 6.662656 | 1.307893 | -0.128447 |
| C | 5.882698 | -2.061898 | -0.645673 |
| H | 5.206283 | -2.816470 | -0.211325 |
| H | 6.912796 | -2.431766 | -0.515645 |

| | | | |
|---|---|---|---|
| H | 5.672480 | -2.005045 | -1.725265 |
| C | 6.010345 | -0.802201 | 1.550915 |
| H | 5.887232 | 0.165127 | 2.062526 |
| H | 7.043991 | -1.148281 | 1.713904 |
| H | 5.341396 | -1.528264 | 2.041859 |
| H | 3.169000 | -1.695275 | 0.813467 |
| H | -2.997190 | -1.590306 | 0.768958 |
| O | 0.389280 | 0.449992 | 2.114106 |
| O | -0.055454 | -1.328477 | -1.440475 |
| N | -0.873967 | -2.359754 | -1.732582 |
| N | 0.156651 | 1.733110 | 2.461716 |
| O | -0.773723 | -2.822248 | -2.868187 |
| O | -1.673651 | -2.767313 | -0.863063 |
| O | 0.798047 | 2.146407 | 3.429758 |
| O | -0.688179 | 2.394158 | 1.830987 |
| N | 0.082415 | -1.947313 | 1.232123 |
| O | 0.130857 | -2.951880 | 1.866864 |

**Table S6a.** Thermodynamics for all species optimized at the BP86/6-31G(d) level of theory

| Species | E(SCF) / $E_h$ | G(gas) / $E_h$ | ΔG(gas) / kcal/mol |
|---|---|---|---|
| A | -2748.148864 | -2747.778725 | 0.00 |
| B | -2748.107381 | -2747.732270 | 29.15 |
| C | -2748.154109 | -2747.782068 | -2.10 |
| D | -2748.157415 | -2747.780757 | -1.28 |
| $H_2LCr(NO_3)_3$ | -2898.549326 | -2898.168291 | – |

**Table S6b.** Thermodynamics for all species optimized at the BP86/6-311G(d) level of theory

| Species | E(SCF) / $E_h$ | G(gas) / $E_h$ | ΔG(gas) / kcal/mol |
|---|---|---|---|
| A | -2748.614204 | -2748.245574 | 0.00 |
| B | -2748.576568 | -2748.200917 | 28.02 |
| C | -2748.621957 | -2748.251673 | -3.83 |
| D | -2748.628874 | -2748.254034 | -5.31 |
| $H_2LCr(NO_3)_3$ | -2899.064002 | -2898.685194 | – |
